# Gut microbiota from NLRP3-deficient mice ameliorates depressive-like behaviors by regulating astrocyte dysfunction via circHIPK2

**DOI:** 10.1186/s40168-019-0733-3

**Published:** 2019-08-22

**Authors:** Yuan Zhang, Rongrong Huang, Mengjing Cheng, Lirui Wang, Jie Chao, Junxu Li, Peng Zheng, Peng Xie, Zhijun Zhang, Honghong Yao

**Affiliations:** 1 Department of Pharmacology, School of Medicine, Southeast University, Nanjing, Jiangsu China; 2 School of Basic Medicine and Clinical Pharmacy, China Pharmaceutical University, Nanjing, Jiangsu China; 3 Department of Physiology, School of Medicine, Southeast University, Nanjing, Jiangsu China; 4 Department of Pharmacology and Toxicology, University at Buffalo, Buffalo, NY USA; 5 Department of Neurology, The First Affiliated Hospital of Chongqing Medical University, Chongqing, China; 6 Department of Neurology of Affiliated ZhongDa Hospital, Institute of Neuropsychiatry of Southeast University, Nanjing, Jiangsu China; 7 Institute of Life Sciences, Key Laboratory of Developmental Genes and Human Disease, Southeast University, Nanjing, Jiangsu China; 8 Co-innovation Center of Neuroregeneration, Nantong University, Nantong, Jiangsu China

**Keywords:** NLPR3, Gut microbiome, circHIPK2, Astrocyte, Depression

## Abstract

**Background:**

Inflammasomes have been found to interact with the gut microbiota, and this effect is associated with depression, but the mechanisms underlying this interaction have not been elucidated in detail.

**Results:**

The locomotor activity of NLRP3 KO mice was significantly greater than that of their WT littermates, while cohousing and transplantation of the NLRP3 KO gut microbiota avoid the effects of NLRP3 KO on the general locomotor activity at baseline. Meanwhile, transplantation of the NLRP3 KO microbiota alleviated the CUS-induced depressive-like behaviors. The compositions of the gut microbiota in NLRP3 KO mice and WT mice were significantly different in terms of the relative abundance of *Firmicutes*, *Proteobacteria*, and *Bacteroidetes*. Fecal microbiota transplantation (FMT) from NLRP3 KO mice significantly ameliorated the depressive-like behavior induced by chronic unpredictable stress (CUS) in recipient mice. Given the correlation between circular RNA HIPK2 (circHIPK2) and depression and the observation that the level of circHIPK2 expression was significantly increased in CUS-treated mice compared with that in the control group, further experiments were performed. FMT significantly ameliorated astrocyte dysfunction in recipient mice treated with CUS via inhibition of circHIPK2 expression.

**Conclusions:**

Our study illustrates the involvement of the gut microbiota-circHIPK2-astrocyte axis in depression, providing translational evidence that transplantation of the gut microbiota from NLRP3 KO mice may serve as a novel therapeutic strategy for depression.

**Electronic supplementary material:**

The online version of this article (10.1186/s40168-019-0733-3) contains supplementary material, which is available to authorized users.

## Background

Major depressive disorder (MDD), which is characterized by emotional dysfunction, is one of the most prevalent psychiatric disorders worldwide [1–4]. MDD arises from a combination of genetic and environmental factors [5], with stress being a major environmental risk factor [6]. Several theories have attempted to explain the pathogenesis of MDD [7–9], but a definitive answer remains elusive. Increasing evidence suggests that gut microbiota is an environmental factor that can shape the brain through the microbiota-gut-brain axis [10, 11]. Mice with an altered microbiota often display depression-related behaviors [12, 13], and the gut microbiota composition is altered in depressive patients [14] and animal models [11, 12, 15]. Transplanting the microbiota from MDD patients into germ-free mice led to depressive behavior [14], and transplanting the gut microbiota from depressive patients into rats subjected to antibiotic treatment also replicated depressive behavior [16]. These results further indicated that alteration of the gut microbiota composition may be an important factor leading to depression. However, the detailed mechanisms by which the microbiota affects depressive-like behaviors have not been determined.

According to the inflammasome hypothesis of depression, neuroinflammatory pathways play a role in MDD [14, 17, 18]. Caspase-1 and NLRP3 mRNA levels are increased in the blood cells of depressed patients [19], suggesting that inflammasomes are a key mediator in the development of depression [20]. Recent evidence also suggests that NLRP3 is a common mediator in the development of depression [17, 21]. NLRP3 has been found to be activated in depression patients [19] and rodent models of depression [22–24]. Intriguingly, caspase-1 KO resulted in decreased depressive-like behavior, and administration of the caspase-1 inhibitor minocycline ameliorated depressive-like behavior by modulating the relationship between stress and the gut microbiota composition [18]. Despite these findings, a detailed understanding of the interactions between the inflammasome and the gut microbiota is still lacking.

Astrocyte dysfunction is known to play a critical role in depression [25] Astrocyte function is affected by factors produced within and outside the central nervous system (CNS) [26]. A previous study demonstrated that microbial metabolites activate aryl hydrocarbon receptor signaling in astrocytes and suppress CNS inflammation [27]. The absence of a complex host microbiota also leads to defects in microglial maturation, differentiation, and function [28], demonstrating that the gut microbiota promotes the maintenance of microglia under steady-state conditions. However, whether fecal microbiota transplantation (FMT) affects astrocyte function is largely unknown.

Circular RNAs (circRNAs) are highly expressed in the brain and are involved in the regulation of physiological and pathophysiological processes. Our previous study indicated that circular RNA HIPK2 (circHIPK2) inhibited astrocyte activation [29]. Given the astrocyte dysfunction in depression and the role of circHIPK2 in astrocyte activation, it is tempting to speculate that changes in the microbiota could partially alter behavior via circHIPK2-regulated astrocyte function in the context of depression.

We tested this hypothesis by comparing the gut microbial communities of WT and NLRP3 KO littermates and evaluating whether alterations in the gut microbiota are associated with depressive-like behaviors. We then assessed how gut microbiota from NLRP3 KO mice influence the behavioral characteristics of mice subjected to chronic unexpected stress (CUS), especially regarding whether the gut microbiota may be a factor contributing to astrocyte function via the regulation of circRNAs.

## Results

### NLRP3 KO gut microbiota affected depressive-like behavior

**Fig. 1:**
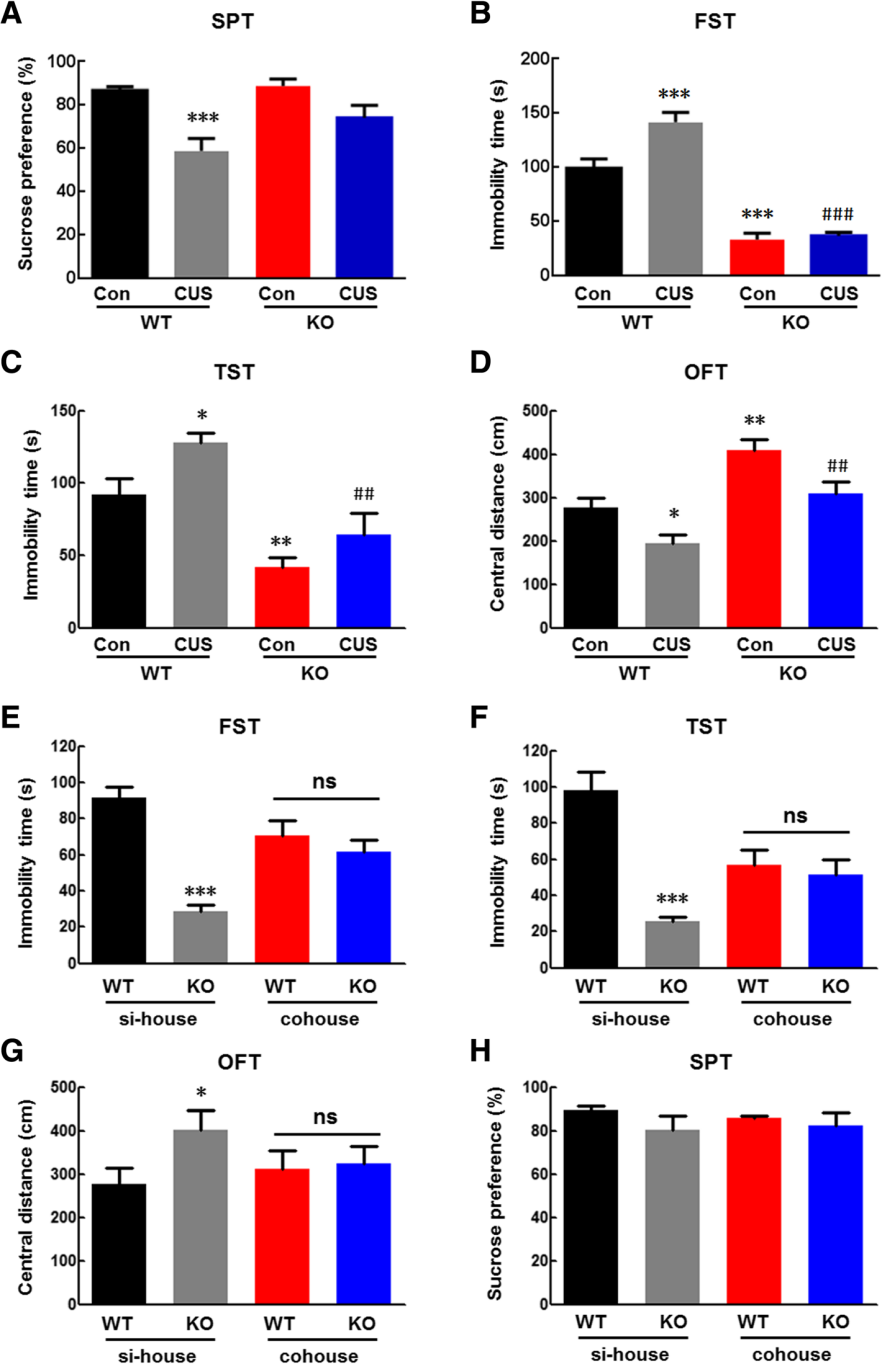
NLRP3 KO gut microbiota affected depressive-like behavior. **a** Compared with WT mice, NLRP3 inflammasome deficiency exerted no significant effect on the sucrose preference induced by CUS. **b**–**d** Compared with WT mice, NLRP3 inflammasome deficiency inhibited the increased immobility time in the FST (**b**) and TST (**c**) induced by CUS. *N* = 6–13 mice/group. *p < 0.05, **p < 0.01 and ***p < 0.001 vs. the WT control group. ^##^*p* < 0.01 and ^###^*p* < 0.001 vs. the CUS-treated WT group using one-way ANOVA followed by the Holm-Sidak test. **d**–**f** Cohousing reduced the significant differences in behavior between WT and NLRP3 KO littermates, as demonstrated by the FST (**d**) and TST (**e**). **f** Cohousing exerted no significant effect on sucrose preference. *N* = 11 mice/group. *p < 0.05 and ***p < 0.001 vs. the si-house-treated WT group using Student’s *t* test. si-house, only WT mice or KO mice were housed in a cage; cohouse, WT and KO mice were housed in a cage

The behaviors of WT and NLRP3 KO littermates were compared. Depression-like behavior was assessed in the sucrose preference test (SPT), forced swim test (FST) and tail suspension test (TST). Locomotor activity and anxiety-like behavior were evaluated in the open field test (OFT). The locomotor activity of NLRP3 KO mice was significantly greater than that of WT littermates, except for the sucrose preference, there were significant differences in behavior between the two groups (Additional file 1: Figure S1A–F). CUS treatment had no effect on locomotor activity (the total distance in the OFT) (Additional file 1: Figure S2A). However, CUS treatment resulted in decreased sucrose preference in the SPT and increased immobility time in the FST and TST, which were inhibited by NLRP3 inflammasome deficiency (Fig. 1a–c). These effects were also observed for the behavior of the time and distance spent exploring the central region in the OFT (Additional file 1: Figure S2B, C). Next, we addressed whether alterations of the NLRP3 KO mouse microbiota directly underlie these different behaviors. We cohoused WT and NLRP3 KO mice at a 1:1 ratio from weaning to adulthood to exchange their microbiota. We found that cohousing prevented the effects of NLRP3 inflammasome deficiency on locomotor activity at baseline (Additional file 1: Figure S3A) but reduced the significant differences in depressive and anxiety-like behaviors between WT and NLRP3 KO littermates as demonstrated by the immobility time in the TST and FST (Fig. 1d, e), and the time and distance spent exploring the central region in the OFT (Additional file 1: Figure S3B, C). As expected, cohousing exerted no significant effect on sucrose preference (Fig. 1f).

### NLRP3 inflammasome deficiency affected the gut microbiota composition

**Fig. 2:**
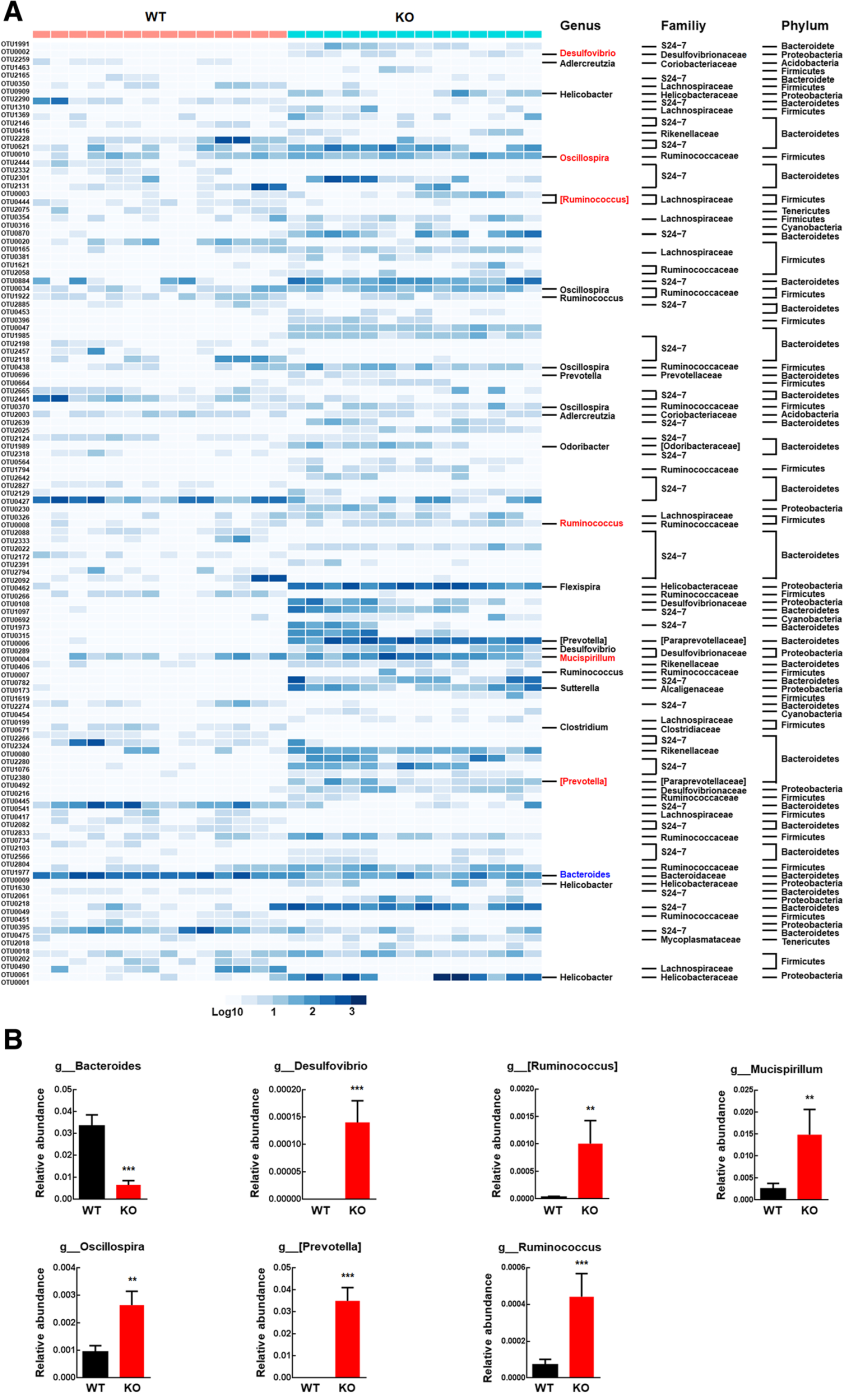
NLRP3 inflammasome deficiency affected the gut microbiota composition. **a** Three-dimensional PCoA of unweighted UniFrac distances showed obvious differences in the gut microbiota composition between WT and NLRP3 KO mice. Heatmap of the 120 discriminative OTUs between WT and NLRP3 KO mice. Each OTU ID and taxonomic assignment is provided to the right of the heatmap. **b** Relative abundances of genera significantly altered by NLRP3 inflammasome deficiency in the gut microbiota. *N* = 14 mice/group. **p < 0.01 and ***p < 0.001 vs. the WT group using the Mann-Whitney test

To investigate whether there was a difference between the gut microbial communities of WT and NLRP3 KO littermates, we employed 16S ribosomal RNA (16S rRNA) gene sequencing. Unweighted UniFrac analysis, which focuses on the degree of microbial phylogenetic similarity, was used to determine the degree to which the gut microbiota in the NLRP3 KO group differed from that in the WT group. Principal coordinate analysis (PCoA) revealed a markedly different microbial landscape between the WT and NLRP3 KO groups (Additional file 1: Figure S4). To identify the component of the gut microbiota primarily responsible for discriminating the two groups, we applied a random forests classifier, which assigns an importance score to each operational taxonomic unit (OTU) by estimating the increase in error caused by removing that OTU from the set of predictors. A total of 120 OTUs whose relative abundance reliably distinguished NLRP3 KO and WT littermates were identified (Fig. 2a). At the phylum level, 74 OTUs were enriched in the gut microbiota of NLRP3 KO mice; among these OTUs, 27 OTUs belonged to the family S24-7, *Rikenellaceae*, *Paraprevotellaceae*, *Prevotellaceae*, or *Odoribacteraceae* of the phylum *Bacteroidetes*; 20 OTUs belonged to the family *Ruminococcaceae* or *Lachnospiraceae* of the phylum *Firmicutes*; 9 OTUs belonged to the family *Desulfovibrionaceae*, Helicobacteraceae, or *Alcaligenaceae* of the phylum *Proteobacteria*; and 18 OTUs belonged to the family *Mycoplasmataceae* or were unclassified (Fig. 2a). In contrast, 46 OTUs were enriched in the gut microbiota of WT mice, among which 33 OTUs belonged to the family S24-7 or *Bacteroidaceae* of the phylum *Bacteroidetes*; 8 OTUs belonged to the family *Ruminococcaceae*, *Lachnospiraceae*, *Coriobacteriaceae*, or *Clostridiaceae* of the phylum *Firmicutes*; and 5 OTUs belonged to the family *Deferribacteraceae* or were unclassified (Fig. 2a). At the genus level, NLRP3 inflammasome deficiency decreased the relative abundances of *Bacteroides* but increased the abundances of *Desulfovibrio*, [*Ruminococcus*], *Mucispirillum*, *Oscillospira*, [*Prevotella*], and *Ruminococcus* (Fig. 2b).

### Transplantation of the NLRP3 KO gut microbiota ameliorated CUS-induced depressive-like behaviors

**Fig. 3:**
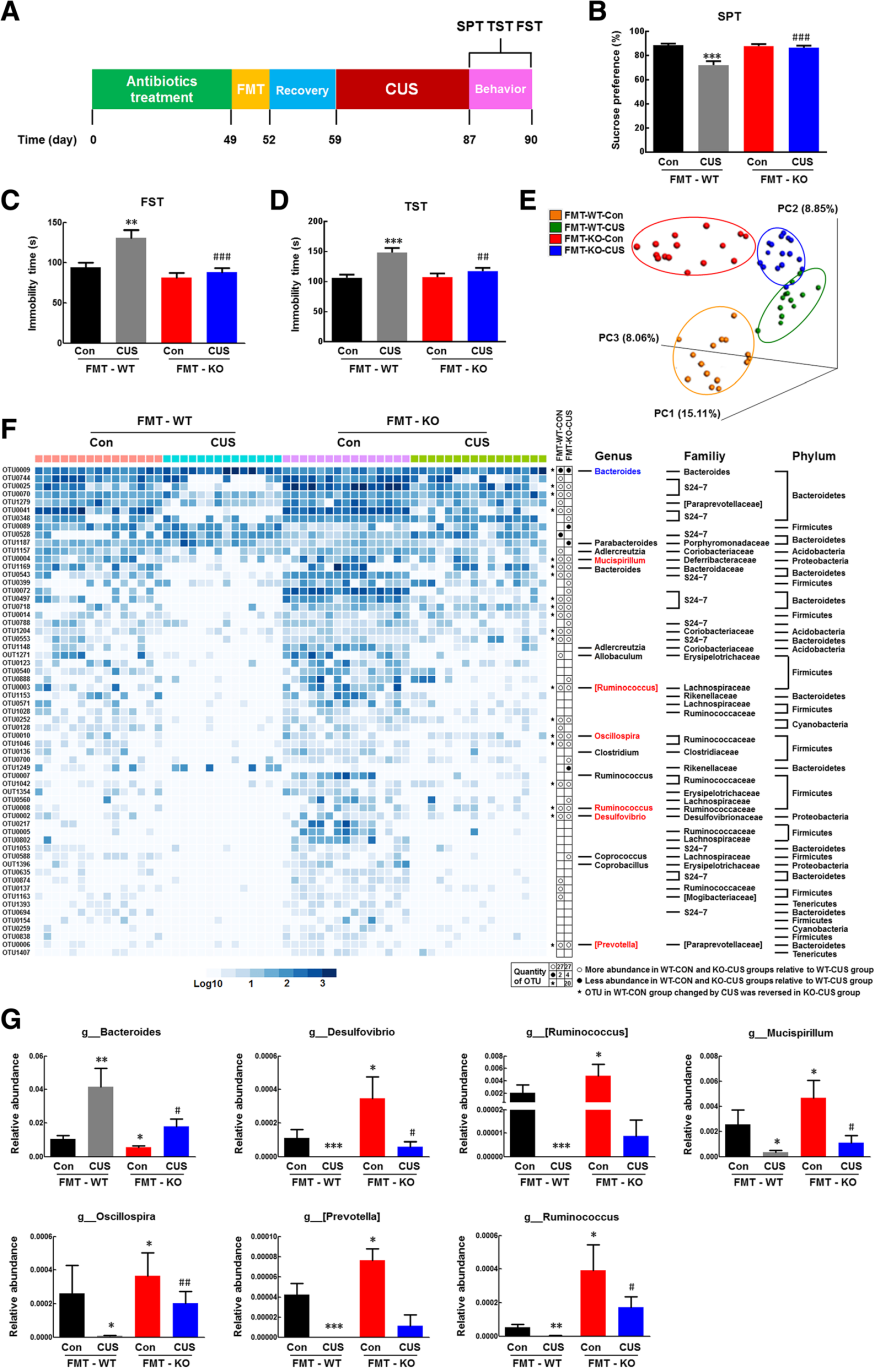
Transplantation of the NLRP3 KO gut microbiota ameliorated CUS-induced depressive-like behaviors. **a** FMT experimental design and behavioral tests. Mice were treated with antibiotics for 7 weeks and gavaged with the fecal contents of either WT or NLRP3 KO donor mice for 3 days. After 1 week of recovery, the FMT recipient mice were subjected to CUS for 4 weeks, and behavioral tests were performed before the mice were sacrificed. **b** Compared to WT microbiota recipient mice, NLRP3 KO microbiota recipient mice displayed an inhibition of the CUS-induced decrease in sucrose preference. **c**, **d** Compared to WT microbiota recipient mice, NLRP3 KO microbiota recipient mice displayed an inhibition of the CUS-induced increase in immobility time in the FST (**c**) and TST (**d**). *N* = 20 mice/group. **p < 0.01 and ***p < 0.001 vs. the FMT-WT control group. ^#^*p* < 0.05, ^##^*p* < 0.01 and ^###^*p* < 0.001 vs. the CUS-treated FMT-WT group using one-way ANOVA followed by the Holm-Sidak test. **e** Three-dimensional PCoA of unweighted UniFrac distances showed obvious differences in the gut microbiota composition between FMT-WT and FMT-NLRP3 KO mice with/without CUS treatment. **f** Heatmap of the 61 discriminative OTUs among FMT-WT and FMT-NLRP3 KO mice with/without CUS treatment. Each OTU ID and taxonomic assignment is provided to the right of the heatmap. Relative abundances of the phyla present in samples from the FMT-WT control group (pink bar), CUS-treated FMT-WT group (blue bar), FMT-NLRP3 KO control group (purple bar), and CUS-treated FMT-NLRP3 KO group (green bar). **g** Mice transplanted with the NLRP3 KO microbiota showed an inhibition of the CUS-induced alteration in the relative abundances of genera compared to WT microbiota recipient mice. *N* = 12-16 mice/group. *p < 0.05, **p < 0.01 and ***p < 0.001 vs. the FMT-WT control group. ^#^*p* < 0.05 and ^##^*p* < 0.01 vs. the CUS-treated FMT-WT group using the Mann-Whitney test

To investigate whether changes in the gut microbiota contribute to the alteration of behaviors in NLRP3 KO mice, we performed FMT experiments, as illustrated in Fig. 3a. Interestingly, the NLRP3 KO microbiota recipient mice avoided the effects of NLRP3 KO on the locomotor activity at baseline (Additional file 1: Figure S5A). The WT microbiota recipient mice displayed a decreased sucrose preference in the SPT and an increased immobility time in the FST and TST after CUS treatment, and these effects were alleviated in the NLRP3 KO microbiota recipient mice (Fig. 3b, d). These effects were also observed for anxiety-like behavior (Additional file 1: Figure S5B, C). These findings suggest that depressive and anxiety-like behaviors were transmissible via the gut microbiota and that colonization with the NLRP3 KO microbiota decreased these behaviors. To determine whether the differences in the gut microbiota between WT and NLRP3 KO mice were maintained in the recipient mice, the microbial communities in the cecum stools harvested from recipient mice treated with or without CUS were subjected to 16S rRNA gene sequencing at 4 weeks post-FMT. PCoA showed a clear difference between the WT microbiota recipient mice and the NLRP3 KO microbiota recipient mice, while similarities were observed between the corresponding donor and recipient mice (Additional file 1: Figure S6). However, after transplantation and CUS treatment, the gut microbiota of each group showed a separation between WT and NLRP3 KO microbiota recipient mice with or without CUS treatment (Fig. 3e). At the phylum level, 61 OTUs were responsible for discriminating the gut microbiota in WT recipient mice treated with CUS. CUS treatment increased the levels of 8 OTUs that belonged to the families S24-7, *Bacteroidaceae*, *Rikenellaceae*, and *Porphyromonadaceae* of the phylum *Bacteroidetes*; the family *Coriobacteriaceae*; or the unclassified. CUS treatment decreased the levels of 19 OTUs belonging to the family S24-7, *Bacteroidaceae*, *Rikenellaceae*, or *Paraprevotellaceae* of the phylum *Bacteroidetes*; 18 OTUs belonging to the family *Ruminococcaceae*, *Lachnospiraceae*, *Erysipelotrichaceae*, *Clostridiaceae*, or *Mogibacteriaceae* of the phylum *Firmicutes*; and 16 OTUs that belonged to the family *Desulfovibrionaceae*, *Deferribacteraceae*, or the unclassified. These disorders of the gut microbiota were ameliorated in the NLRP3 KO microbiota recipient mice (Fig. 3f).

At the genus level, NLRP3 KO microbiota recipient mice exhibited a decrease in the relative abundance of *Bacteroides* and increases in that abundances of *Desulfovibrio*, [*Ruminococcus*], *Mucispirillum*, *Oscillospira*, [*Prevotella*], and *Ruminococcus* compared to those in WT microbiota recipient mice (Fig. 3g). These changes were consistent with observations in NLRP3 KO mice relative to WT mice (Fig. 2b). Moreover, mice transplanted with the NLRP3 KO microbiota exhibited reductions in the CUS-induced changes in *Bacteroides*, *Desulfovibrio*, [*Ruminococcus*], *Mucispirillum*, *Oscillospira*, [*Prevotella*], and *Ruminococcus* compared to WT microbiota recipient mice (Fig. 3g), indicating that NLRP3 inflammasome deficiency inhibits the change in depressive-like behavior by remodeling the gut microbiota composition.

### Transplantation of the gut microbiota from NLRP3 KO mice alleviated astrocyte dysfunction in CUS mice

**Fig. 4:**
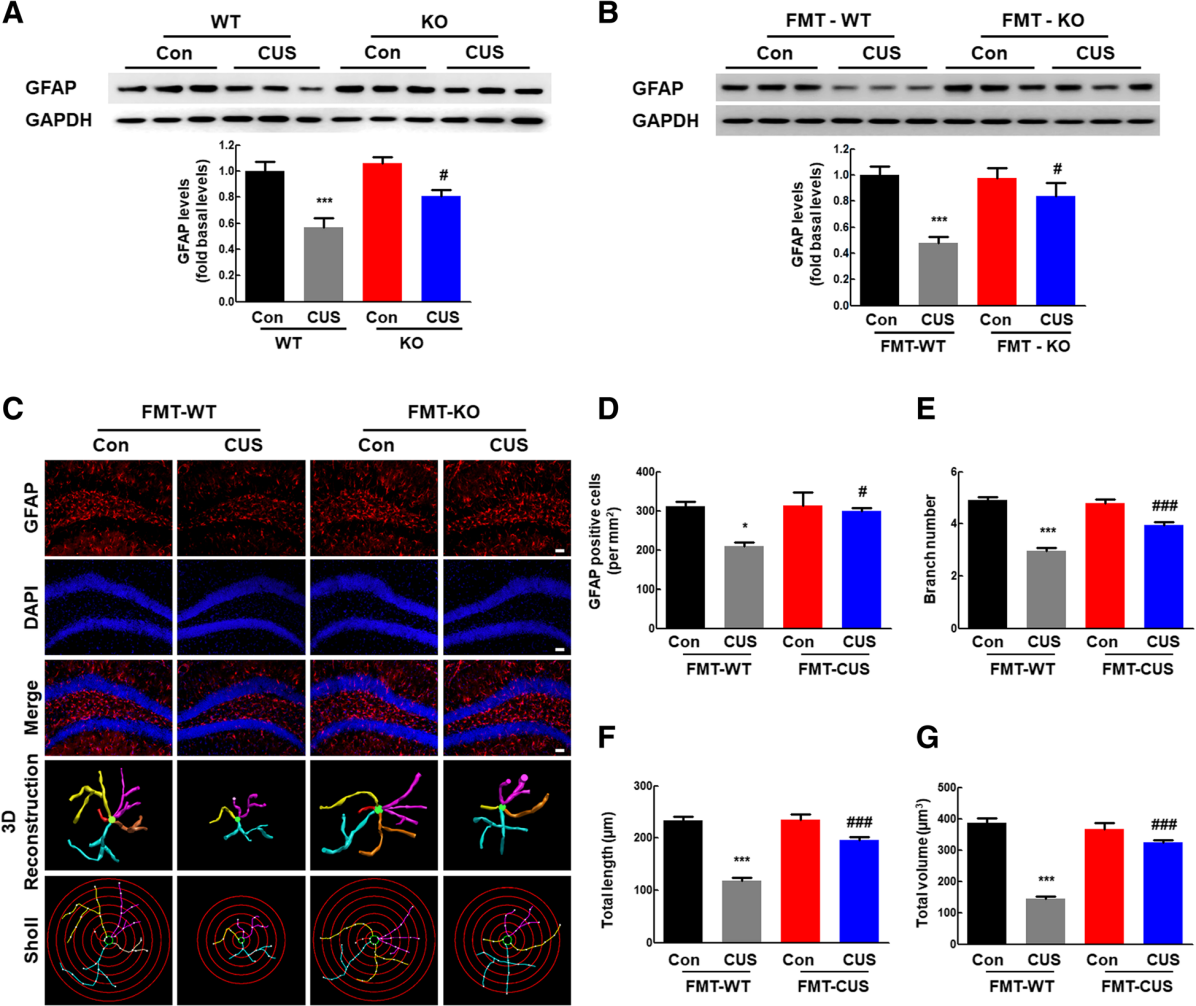
Transplantation of the gut microbiota from NLRP3 KO mice alleviated astrocyte dysfunction in CUS mice. **a** NLRP3 inflammasome deficiency rescued the decreased GFAP levels induced by CUS. *N* = 6 mice/group. ***p < 0.001 vs. the WT control group. ^#^*p* < 0.05 vs. the CUS-treated WT group using one-way ANOVA followed by the Holm-Sidak test. **b** Colonization with the NLRP3 KO microbiota attenuated the decreased GFAP expression induced by CUS. *N* = 11 mice/group. **c** Effect of NLRP3 KO microbiota colonization on astrocyte dysfunction induced by CUS. Representative images of astrocyte immunostaining for GFAP in mouse hippocampi, followed by 3D reconstruction and Sholl analysis. Scale bars, 50 μm. **d** Quantification of GFAP-positive cells per square millimeter in mouse hippocampi. *N* = 4 mice/group. **e**–**g** Average branch number (**e**), total branch length (**f**), and total branch volume (**g**). *n* = 4 mice/group, 40 cells/group. *p < 0.05 and ***p < 0.001 vs. the FMT-WT control group. ^#^*p* < 0.05 and ^###^*p* < 0.001 vs. the CUS-treated FMT-WT group using one-way ANOVA followed by the Holm-Sidak test.

Since astrocyte dysfunction was found to be involved in depression and antibiotic cocktail treatment did not influence on astrocyte activation (Additional file 1: Figure S7), we next examined the effect of transplantation of the gut microbiota from NLRP3 KO mice on astrocyte function. As shown in Fig. 4a, CUS treatment resulted in astrocyte dysfunction, which was significantly inhibited in NLRP3 KO littermates. Astrocyte dysfunction induced by CUS was also found in WT microbiota recipient mice, while colonization with the NLRP3 KO microbiota attenuated CUS-induced astrocyte dysfunction (Fig. 4b). This finding was confirmed by GFAP staining (Fig. 4c). CUS exposure resulted in astrocyte dysfunction in WT microbiota recipient mice, as indicated by the fact that CUS exposure decreased the number of GFAP-positive cells (Fig. 4d) and the ramification of astrocytes, as characterized by significantly decreased branch numbers, length, and volume (Fig. 4e–f). These effects were significantly attenuated by colonization with the NLRP3 KO microbiota. These findings suggest that colonization with the NLRP3 KO microbiota significantly ameliorated astrocyte dysfunction in CUS mice.

### Transplantation of the NLRP3 KO gut microbiota inhibited the increased expression of circHIPK2 in CUS mice

**Fig. 5:**
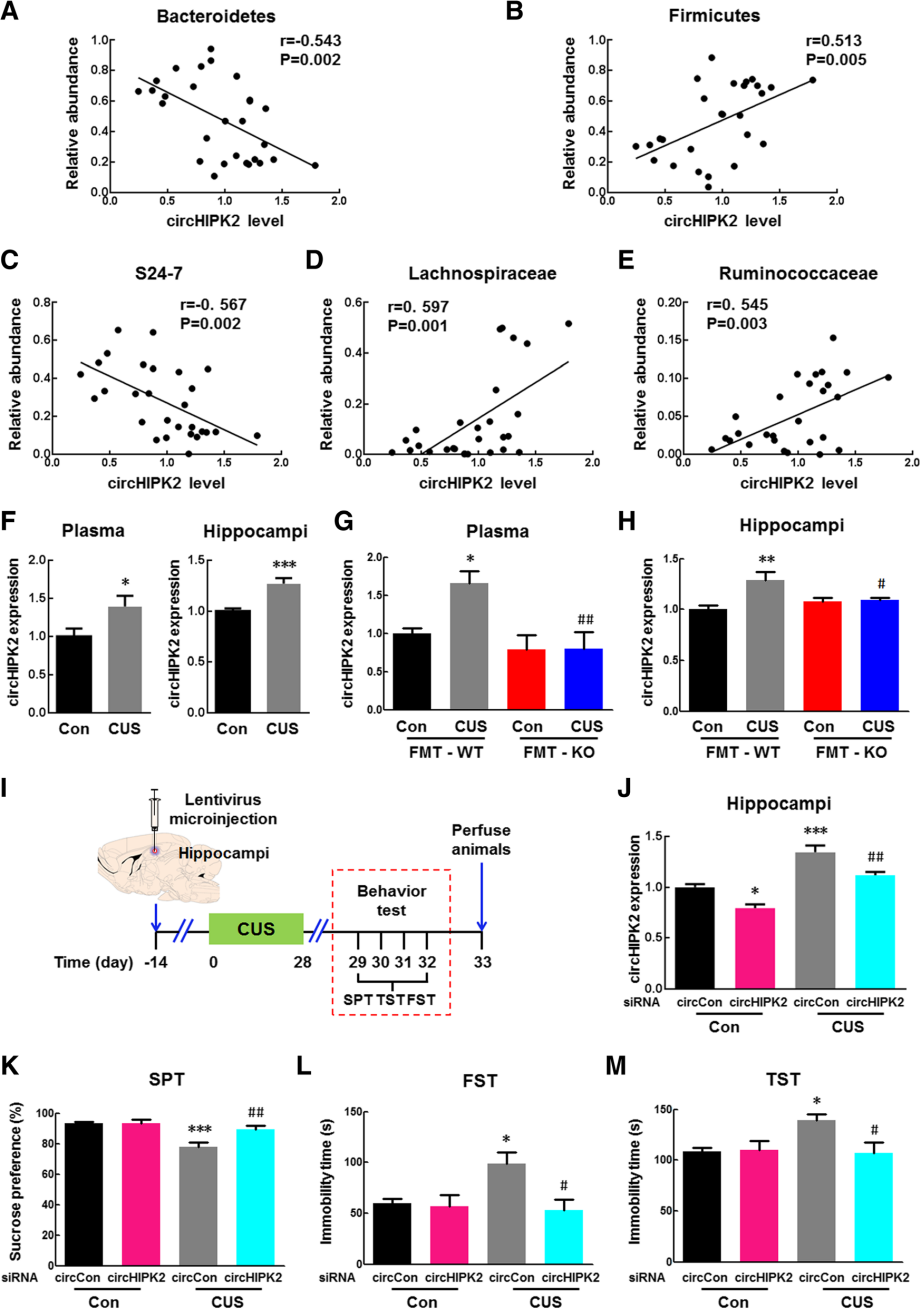
Transplantation of the NLRP3 KO gut microbiota inhibited the increased expression of circHIPK2 in CUS mice. **a–e** The correlation of the relative abundances of phyla (*Bacteroidetes* and *Firmicutes*) and families (S24-7, *Ruminococcaceae* and *Lachnospiraceae*) with circHIPK2 levels in the plasma of CUS-treated mice. **f** circHIPK2 levels in plasma and hippocampi were significantly increased by CUS treatment. *N* = 9–12 mice/group. *p < 0.05 and ***p < 0.001 vs. the control group using Student’s *t* test. **g**, **h** circHIPK2 levels in the plasma (**g**) and hippocampi (**h**) of the WT microbiota recipient mice were significantly increased by CUS treatment, and these levels were significantly inhibited in the NLRP3 KO microbiota recipient mice. *N* = 6–8 mice/group. *p < 0.05 and **p < 0.01 vs. the FMT-WT control group. ^#^*p* < 0.05, and ^##^*p* < 0.01 vs. the CUS-treated FMT-WT group using one-way ANOVA followed by the Holm-Sidak test. **i** Illustration of lentivirus microinjection and the experimental procedure. Mice were microinjected with the GFP-labeled circCon or circHIPK2 siRNA lentivirus for 2 weeks, followed by CUS treatment for another 4 weeks. Behavioral tests were performed before the mice were sacrificed. **j** circHIPK2 levels decreased in circHIPK2 siRNA-injected mice compared with those in circ siRNA control-injected mice in both the control and CUS-treated groups. **k** circHIPK2 siRNA microinjection significantly attenuated the CUS-induced decrease in sucrose preference. **l**, **m** circHIPK2 siRNA microinjection significantly inhibited the CUS-induced increase in immobility time in the FST (**l**) and TST (**m**). *N* = 7–15 mice/group. *p < 0.05, **p < 0.01 and ***p < 0.001 vs. the circCon control group. ^#^*p* < 0.05 and ^##^*p* < 0.01 vs. the CUS-treated circCon group using one-way ANOVA followed by the Holm-Sidak test.

Our previous work demonstrated that silencing circHIPK2 inhibited astrocyte activation induced by lipopolysaccharide (LPS) [29]. Meanwhile, antibiotic cocktail treatment did not exert a significant effect on the circHIPK2 expression (Additional file 1: Figure S8). Therefore, we examined whether circHIPK2 lies downstream of the NLRP3 KO gut microbiota to regulate astrocyte function in mice. We first examined whether there is a correlation between the relative abundances of bacteria and circHIPK2 levels in the plasma of CUS-treated mice. At the phylum level, we identified that *Bacteroidetes* was negatively correlated with the circHIPK2 level, while *Firmicutes* showed a positive correlation (Fig. 5a, b). At the family level, the abundance of S24-7 was found to have a significant negative correlation with the circHIPK2 level. Conversely, the relative abundance of *Ruminococcaceae* and *Lachnospiraceae* had a significant positive correlation with the circHIPK2 level (Fig. 5c–e). Next, we examined circHIPK2 levels in mice treated with or without CUS. As shown in Fig. 5f, circHIPK2 levels were significantly increased by CUS treatment in the plasma and hippocampi. However, there was no significant difference in circHIPK2 expression in other brain regions, such as the cortex, amygdala, and hypothalamus (Additional file 1: Figure S9). We then examined circHIPK2 levels in the plasma and hippocampi of microbiota recipient mice. As shown in Fig. 5g, h, the levels of circHIPK2 in WT microbiota recipient mice were significantly increased by CUS treatment, and this effect was inhibited in NLRP3 KO microbiota recipient mice.

Having determined that the gut microbiota composition affected circHIPK2 levels, we next examined whether circHIPK2 plays a critical role in animal behaviors in vivo by microinjecting a circHIPK2 siRNA lentivirus into the hippocampi of mice. One week after the lentivirus microinjection, the mice were treated with CUS, and the behaviors were assessed by the SPT, FST, TST, and OFT (Fig. 5i). As expected, decreased circHIPK2 expression was observed in circHIPK2 siRNA-injected mice compared with that in siRNA circ-control-injected mice (Fig. 5j). circHIPK2 siRNA-injected mice displayed no effect on the locomotor activity of mice (Additional file 1: Figure S10A). Compared with control mice, CUS-treated mice displayed decreased sucrose preference, increased immobility time (Fig. 5k–m), and decreased time and distance traveled in the central area (Additional file 1: Figure S10B, C); however, these effects were significantly attenuated by circHIPK2 siRNA microinjection. These findings suggest that knockdown of circHIPK2 expression significantly inhibits depressive and anxiety-like behaviors induced by CUS.

### Knockdown of circHIPK2 expression reversed astrocyte dysfunction induced by CUS

**Fig. 6:**
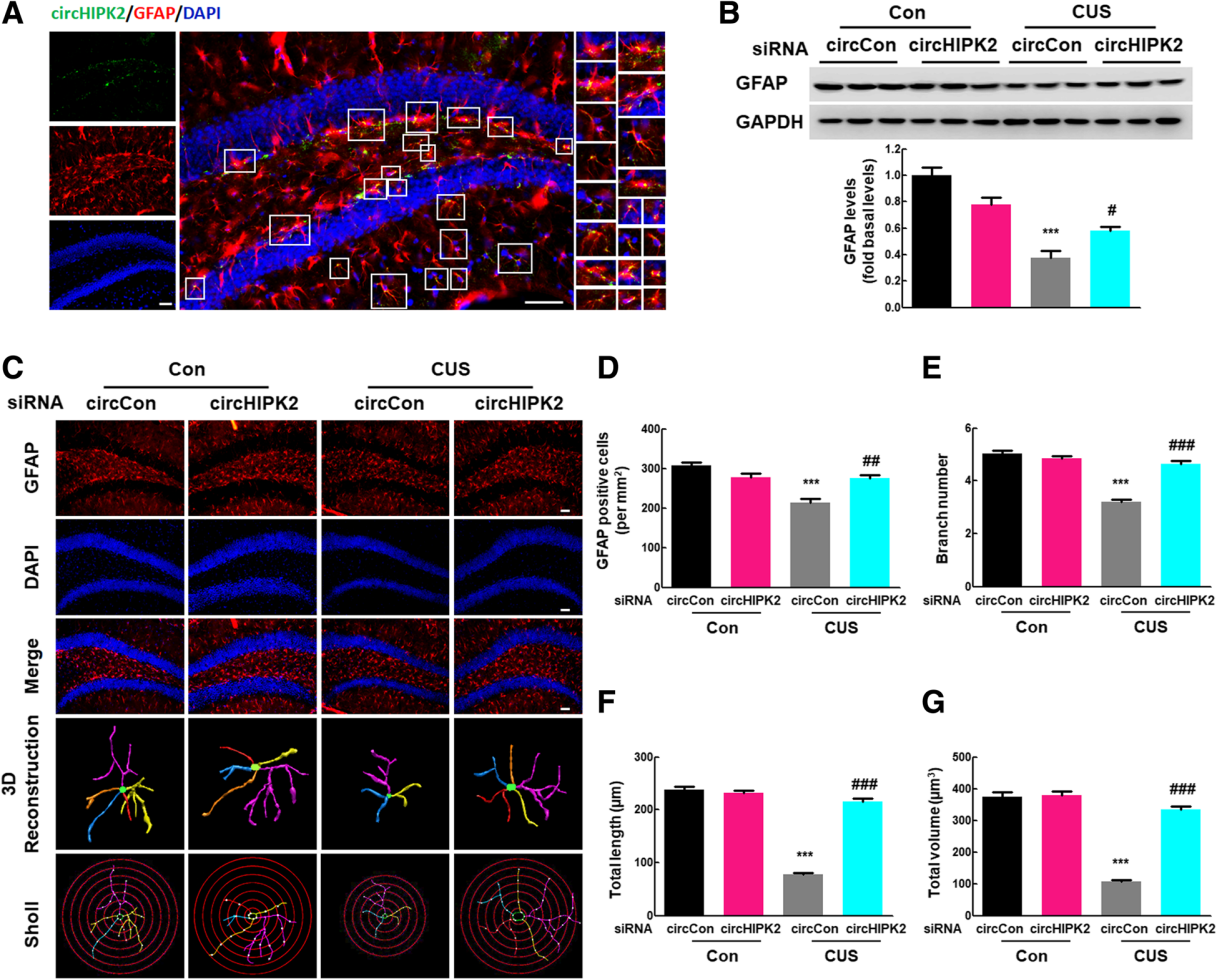
Knockdown of circHIPK2 expression alleviated the astrocyte dysfunction induced by CUS. **a** Colocalization of circHIPK2 and GFAP-positive cells in mouse hippocampi. Green represents circHIPK2; red, GFAP; blue, DAPI. Scale bar, 10 μm. **b** circHIPK2 siRNA microinjection significantly attenuated CUS-induced decreased GFAP expression. Mice were microinjected with the circCon or circHIPK2 siRNA lentivirus followed by CUS treatment. *N* = 6 mice/group. **c** Effect of circHIPK2 knockdown on astrocyte dysfunction induced by CUS. Representative images of astrocyte immunostaining for GFAP in mouse hippocampi, followed by 3D reconstruction and Sholl analysis. Scale bars, 50 μm. **d** Quantification of GFAP-positive cells per square millimeter in mouse hippocampi. *N* = 4 mice/group. **e**–**g** Average branch number (**e**), total branch length (**f**), and total branch volume (**g**). *n* = 4 mice/group, 40 cells/group. ***p < 0.001 vs. the circCon control group. ^#^*p* < 0.05, ^##^*p* < 0.01 and ^###^*p* < 0.001 vs. the CUS-treated circCon group using one-way ANOVA followed by the Holm-Sidak test.

Next, we examined the effect of circHIPK2 on astrocyte function and astrocyte dysfunction induced by CUS treatment. As shown in Fig. 6a, in situ hybridization confirmed the colocalization of circHIPK2 and GFAP-positive cells in vivo in mouse hippocampi. CircHIPK2 siRNA microinjection significantly inhibited the CUS-induced decrease in GFAP expression (Fig. 6b), and this effect was confirmed by GFAP staining (Fig. 6c). CUS exposure resulted in astrocyte dysfunction, as indicated by the fact that CUS exposure decreased the number of GFAP-positive cells (Fig. 6d) and the ramification of astrocytes, as characterized by significantly decreased branch numbers, length, and volume (Fig. 6e–g). These effects were significantly attenuated by circHIPK2 microinjection. These findings suggest that the knockdown of circHIPK2 expression significantly ameliorated astrocyte dysfunction in CUS mice.

### Knockdown of circHIPK2 expression in astrocytes inhibited depressive-like behavior induced by CUS

**Fig. 7:**
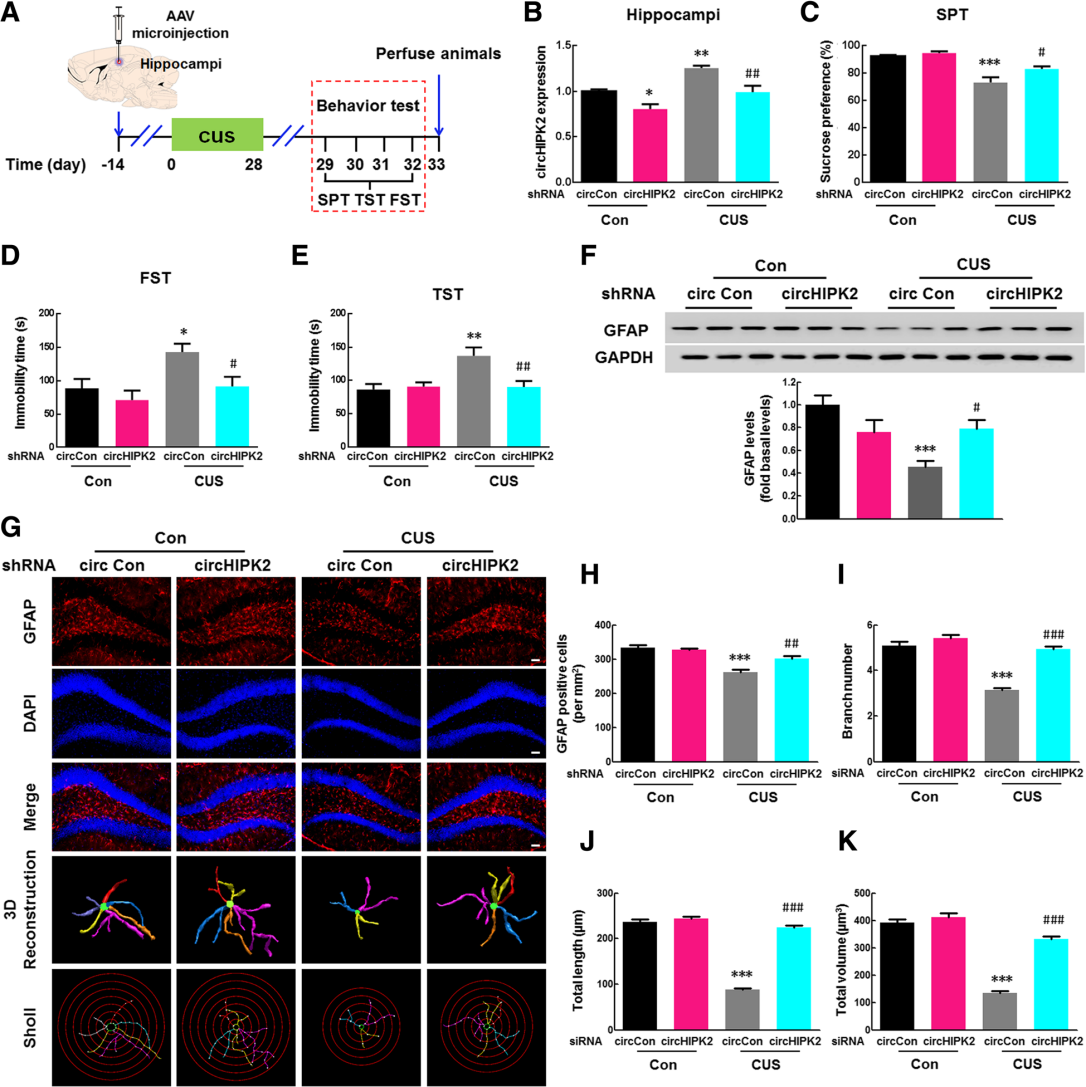
Knockdown of circHIPK2 expression in astrocytes ameliorated the depressive-like behavior induced by CUS. **a** Illustration of AAV microinjection and the experimental procedure. Mice were microinjected with the eGFP-labeled circCon or circHIPK2 shRNA AAV for 2 weeks, followed by CUS treatment for 4 weeks. **b** circHIPK2 levels decreased in circHIPK2 shRNA AAV-injected mice compared with those in circCon shRNA AAV-injected mice in both the control and CUS-treated groups. **c** Specific circHIPK2 expression knockdown in astrocytes attenuated the CUS-induced decrease in sucrose preference. **d**, **e** Specific circHIPK2 expression knockdown in astrocytes inhibited the CUS-induced increase in immobility time in the FST (**d**) and TST (**e**). *N* = 7–15 mice/group. **f** Specific circHIPK2 expression knockdown in astrocytes attenuated the CUS-induced decrease in GFAP expression. *N* = 6 mice/group. **g** Specific knockdown of circHIPK2 expression in astrocytes attenuated the astrocyte dysfunction induced by CUS. Representative images of astrocyte immunostaining for GFAP in mouse hippocampi, followed by 3D reconstruction and Sholl analysis. Scale bars, 50 μm. **h** Quantification of GFAP-positive cells per square millimeter^2^in mouse hippocampi. *N* = 4 mice/group. **i**–**k** Average branch number (**i**), total branch length (**j**), and total branch volume (**k**). *n* = 4 mice/group, 40 cells/group. *p < 0.05, **p < 0.01 and ***p < 0.001 vs. the circCon control group. ^#^*p* < 0.05, ^##^*p* < 0.01 and ^###^*p* < 0.001 vs. the CUS-treated circCon group using one-way ANOVA followed by the Holm-Sidak test.

Using a circHIPK2 shRNA adeno-associated virus (AAV) that targets astrocytes, we next tried to identify the role of circHIPK2 in astrocyte function (Additional file 1: Figure S11A). The AAVs were microinjected into the hippocampi of mice and specifically knocked down circHIPK2 expression in astrocytes. The green fluorescence of circHIPK2 shRNA AAV was found to colocalize with astrocytes in hippocampi (Additional file 1: Figure S11B). One week after AAV microinjection, the mice were treated with CUS, and the behaviors were assessed by the SPT, FST, TST, and OFT as shown in Fig. 7a. As expected, decreased circHIPK2 expression was observed in circHIPK2 shRNA AAV-injected mice compared with that in shRNA circ-control-injected mice (Fig. 7b). The microinjection of circHIPK2 shRNA AAV was also found to have no effect on the locomotor activity of mice (Additional file 1: Figure S12A). CUS-treated mice displayed increased depressive (Fig. 7c–e) and anxiety-like behaviors (Additional file 1: Figure S12B, C) compared with control mice, and these effects were significantly attenuated by circHIPK2 expression knockdown in astrocytes. In addition, circHIPK2 shRNA AAV microinjection significantly inhibited the CUS-induced decrease in GFAP expression (Fig. 7f), and this effect was confirmed by GFAP staining (Fig. 7g). CUS exposure resulted in astrocyte dysfunction, as indicated by the fact that CUS exposure decreased the number of GFAP-positive cells (Fig. 7h) and the ramification of astrocytes, as characterized by significantly decreased branch numbers, length, and volume; these effects were significantly attenuated by circHIPK2 shRNA AAV microinjection (Fig. 7i–k). These findings suggest that knockdown of circHIPK2 expression in astrocytes inhibited CUS-induced depressive-like behaviors in mice by alleviating astrocyte dysfunction.

To dissect the relationship between the gut microbiota and circHIPK2-regulation of astrocyte function, we performed a metabolomics analysis using liquid chromatography-mass spectrometry as shown in Additional file 1: Figure S13. Transplantation of the NLRP3 KO microbiota significantly reduced the increase of 24 metabolites induced by CUS. Meanwhile, NLRP3 KO microbiota transplantation increased the level of 27 metabolites in FMT-KO-CUS group compared with FMT-WT-CUS group. These findings suggest that metabolites may be involved in the circHIPK2-mediated regulation of astrocyte function.

Furthermore, we also examined the effect of exogenous peripheral administration of circHIPK2 on CUS behavior. As shown in Additional file 1: Figure S14A, the intravenous injection of circHIPK2 could not affect the expression of circHIPK2 in the brain. This finding may be explained by the fact that in the normal physiological condition, circulating circHIPK2 was not able to reach the brain parenchyma through the intact blood-brain barrier. Meanwhile, intravenous injection of circHIPK2 did not affect depressive-like behavior or astrocyte function compared with the circ-control (Additional file 1: Figure S14B–D). Based upon these findings, it is possible that the expression of circHIPK2 was increased in the hippocampus of the brain, with subsequent release of circHIPK2 into the circulatory system in the context of MDD. Therefore, intravenous injection of circHIPK2 did not lead to depressive-like behavior or astrocyte activation.

## Discussion

In this study, we demonstrated that NLRP3 KO mice exhibited significant difference of behaviors compared with WT mice and that the composition of their gut microbiota was significantly altered. Transplantation of the gut microbiota from NLRP3 KO mice avoid the effects of NLRP3 KO on the general locomotor activity at baseline and significantly ameliorated the depressive-like behavior induced by CUS. The mechanism underlying this process was the regulation of astrocyte dysfunction via circHIPK2 (Additional file 1: Figure S15). These results highlight the gut microbiota as a potential causative factor in depression through its impact on astrocyte regulation.

Our findings support previous studies on the role of the microbiota-gut-brain axis in the regulation of brain function [30–32]. Previous studies also indicated that NLRP3 KO mice displayed decreased anxiety and anhedonic behaviors under basal, unstressed conditions and were resilient to the behavioral deficits caused by CUS exposure [21]. To the best of our knowledge, this study is the first to demonstrate that different gut microbiota compositions contribute to these different depressive-like behaviors. Consistent with our findings, caspase-1 KO mice showed decreased depressive-like behavior at baseline, and administration of the caspase-1 inhibitor minocycline ameliorated the depressive-like behavior induced by chronic restraint stress by modulating the relationship between stress and the gut microbiota composition [18].

The gut microbiota composition of mice subjected to NLRP3 inflammasome deficiency was significantly altered compared with that of control mice. Major differences between the NLRP3 KO and WT groups were observed in the phyla *Firmicutes* and *Bacteroidetes*. *Firmicutes* was significantly increased, whereas *Bacteroidetes* was markedly decreased in the NLRP3 KO group. At the family level, *Lachnospiraceae*, *Ruminococcaceae*, and *Prevotellaceae* were increased in the NLRP3 KO group. These findings fit well with evidence that intestinal microbiome alteration in MDD patients is characterized by significant decreases in the families *Lachnospiraceae*, *Ruminococcaceae*, and *Prevotellaceae* [15]. Consistent with previous human studies [15, 33], *Porphyromonadaceae* and *Rikenellaceae* were principally increased in CUS-treated WT microbiota recipient mice, while *Ruminococcaceae*, *Lachnospiraceae*, *Erysipelotrichaceae*, and *Bacteroidetes* decreased in CUS-treated NLRP3 KO microbiota recipient mice. Transplantation of the gut microbiota from NLRP3 KO mice ameliorated these gut microbiota disorders, thereby ameliorating CUS-induced depressive-like behaviors. In particular, the discriminative OTUs belonged to the genera *Bacteroides*, *Desulfovibrio*, [*Ruminococcus*], *Mucispirillum*, *Oscillospira*, [*Prevotella*], and *Ruminococcus*, which were altered by CUS treatment and reversed by transplantation of the gut microbiota from NLRP3 KO mice. Consistent with these findings in NLRP3 KO mice, the same relative abundance changes in *Desulfovibrio*, [*Ruminococcus*], *Mucispirillum*, *Oscillospira*, [*Prevotella*], and *Ruminococcus* were found in caspase-1 KO littermates (Additional file 1: Figure S16). To the best of our knowledge, this study is the first to dissect the gut microbiota in NLRP3 KO mice, lending credence to the previous concept that inflammasomes contribute to the regulation of depression by the gut microbiota. Further study will be required to dissect the specific components of the microbiota that contribute to depressive-like behaviors. Moreover, modulation of the gut microbiota is remarkably complex and poorly understood and how NLRP3 deficiency modulates the gut microbiota warrants further study.

Another novel finding of our study is that the gut microbiota-circHIPK2 axis integrates the gut microbiota and environmental cues to regulate astrocyte activity. Astrocytes are the most abundant and versatile cells in the brain, participating in most, if not all, brain functions as both a passive housekeeper and an active player [26]. Evidence from clinical, preclinical, and postmortem studies has revealed that depressive-like conditions are associated with a decrease in the number or density of astrocytes and their function [34]. Consistent with these findings, our study showed that CUS treatment decreased GFAP expression, which was ameliorated by circHIPK2 knockdown. However, our previous study demonstrated that the knockdown of circHIPK2 expression significantly decreased the increased GFAP expression induced by LPS treatment [29]. We have demonstrated that circHIPK2 functions as an endogenous miR-124 sponge to sequester miR-124 and inhibit its activity, resulting in increased sigma-receptor 1 expression [29]. In that case, there was a slightly increased expression of GFAP at one week after LPS treatment. Consistently, the current study also showed dynamic GFAP expression during the progression of the depression animal model. As demonstrated in Additional file 1: Figure S17, GFAP levels were measured in the hippocampi of mice treated with CUS for 3 days, 1 week, 2 weeks, 3 weeks, and 4 weeks (Additional file 1: Figure S17A). GFAP expression first increased at 3 days post-CUS and then decreased at 4 weeks post-CUS (Additional file 1: Figure S17B). GFAP expression was confirmed by immunostaining (Additional file 1: Figure S17C). A previous study also reported that there was a trend of increased GFAP expression at day 3, with the peak time at day 7, followed by decreased GFAP expression [35]. Taking another pharmaceutical molecule as an example, the common anti-depression medicine fluoxetine strongly suppressed astrocyte activation in an APP/PS1 mouse model [36]. Interestingly, fluoxetine showed an enormous protective effect on astrocytes and rescued the decreased GFAP expression in the hippocampi of CUS mice [37]. There is a striking resemblance between circHIPK2 siRNA and fluoxetine, which exhibit seemingly opposite effects on astrocytes, and both of these factors play a positive role in astrocyte-relative disease. Taking all results into account, it is possible that circHIPK2 siRNA ameliorates astrocyte dysfunction by inhibiting astrocyte activation, as does fluoxetine. Based upon these findings, it is possible that during the progression of depression, early activation of astrocytes leads to subsequent astrocyte dysfunction. Therefore, microinjection of circHIPK2 siRNA decreased the increased expression of GFAP, which then ameliorated activation-induced astrocyte dysfunction. To the best of our knowledge, this study is the first to elucidate the dynamic responses of astrocytes in depression, which also explains the seemingly paradoxical effect of circHIPK2 on GFAP expression.

The relationship between the gut microbiota and circHIPK2-regulation of astrocyte function were dissected by metabolomics analysis. Transplantation of the NLRP3 KO microbiota significantly reduced the increase of gluconolactone (no. HMDB00150) induced by CUS (Additional file 1: Figure S13). Based upon the fact that low level of gluconolactone was able to increase astrocytes survival via inhibition of ATP depletion [38], it is possible, at least in part, for gluconolactone to ameliorate the astrocyte dysfunction after transplantation of NLRP3 KO microbiota. Meanwhile, NLRP3 KO microbiota transplantation increased the level of berberine (no. HMDB03409) in FMT-KO-CUS group compared with FMT-WT-CUS group (Additional file 1: Figure S13), which exerted neuroprotective function via inhibiting inflammation in astrocyte [39]. The increased level of berberine may inhibit astrocyte activation resulting in the amelioration of the activation-induced astrocyte dysfunction. Therefore, we anticipate that metabolites are involved in the circHIPK2-mediated regulation of astrocyte function. It is possible for metabolites to mediate circHIPK2 expression in the hippocampus of the brain, the detailed mechanisms underlying the interaction between metabolite and circHIPK2 will be investigated in further study.

More complete understanding of gut microbiota-brain communication is required to develop promising microbiota-based therapeutic interventions for neurological disorders. Several mechanisms have been proposed to mediate the communication between the commensal gut microbiota and the brain: (1) communication is enabled by neuronal circuit signals, (2) the brain is influenced by immune responses within the gut or elsewhere, and (3) the brain is directly influenced by microbiota-derived metabolites [10, 40–42]. The present study suggests a new signaling pathway in which astrocyte circHIPK2 was activated by microbial products. This pathway is supported by the following observations: (1) transplantation of the NLRP3 KO microbiota inhibited CUS-induced depressive-like behavior; (2) astrocyte dysfunction induced by CUS was found in WT microbiota recipient mice, while colonization with the NLRP3 KO microbiota attenuated CUS-induced astrocyte dysfunction; (3) circHIPK2 levels in the plasma and hippocampi of WT microbiota recipient mice were significantly increased by CUS treatment, but there was no effect on NLRP3 KO microbiota recipient mice; and (4) knockdown of circHIPK2 expression ameliorated depressive-like behaviors induced by CUS treatment. Future studies are warranted to elucidate the detailed mechanisms by which the microbiota of NLRP3 KO mice regulates circHIPK2 expression.

## Conclusions

Our results reveal a new mechanism of host-microbiota interaction, in which transplantation of the gut microbiota from NLRP3 KO mice ameliorated depressive-like behaviors through the regulation of astrocyte dysfunction via circHIPK2. This study helps to elucidate the interactions between the gut microbiota and circRNAs, providing a basis for future clinical studies of microbiota manipulation and transplantation.

## Methods and materials

### Reagents

The circ-control siRNA-GFP lentivirus and circHIPK2 siRNA-GFP lentivirus based on the sequence 5′-UACCGGUAUGGCCUCACAUTT-3′ were purchased from HANBIO (Shanghai, China). The circ-control shRNA-eGFP AAV and circHIPK2 shRNA-eGFP AAV based on the sequence 5′-UACCGGUAUGGCCUCACAUTT-3′ were obtained from OBIO (Shanghai, China). TRIzol® reagent was purchased from TAKARA BIO INC (9109, Kusatsu, Shiga, Japan). HiScript Q RT SuperMix for qPCR Kit (R123-01) and AceQ qPCR SYBR Green Master Mix (High ROX Premixed) (Q141-02) were purchased from Vazyme Biotech (Nanjing, China). Oligonucleotide primers for real-time polymerase chain reaction (PCR) were synthesized by Invitrogen (Shanghai, China)

### Animals

The NLRP3 KO mice were kindly shared by Dr. Jurg Tschopp from University of Lausanne and Dr. Rongbin Zhou from Institute of Immunology and the CAS Key Laboratory of Innate Immunity and Chronic Disease, School of Life Sciences and Medical Center, University of Science and Technology of China. NLRP3 KO mice on the C57BL/6 background were described previously [43]. C57BL/6J mice (male, 6–8 weeks) were purchased from the Model Animal Research Center of Nanjing University (Nanjing, China). More details were described in Additional file 1: Methods and Materials.

### Antibiotic treatment

C57BL/6J mice (male, 6–8 weeks) were treated with antibiotics according to the previously published protocol [44]. Briefly, the antibiotic compounds were applied via drinking water for 7 weeks and consisted of ampicillin (1 g/l, Meryer, Shanghai, China), vancomycin (500 mg/l, Macklin, Shanghai, China), ciprofloxacin (200 mg/l, Macklin, Shanghai, China), imipenem plus cilastatin (250 mg/l, MSD, Kenilworth, NJ, USA), and metronidazol (1 g/l, Aladdin, Shanghai, China). Antibiotic water bottles were inverted every day, and antibiotic solution was changed every 2–3 days.

### FMT

Fresh fecal transplants were pooled from WT and NLRP3 KO donor mice, respectively. Antibiotic-treated mice were orally challenged with 300 μl fecal transplants (approximately 2 × 10^8^ viable probiotic bacteria dissolved in sterile PBS) by gavaging on 3 consecutive days. The mice were kept on sterile tap water for 7 days recovery after fecal microbial transplantation until CUS induction.

### Microinjection of circHIPK2 siRNA lentivirus or AAV

C57/BL6 mice (6–8 weeks) were microinjected with either the circ-control/circHIPK2 siRNA-GFP lentivirus or the circ-control/circHIPK2 shRNA-eGFP AAV that targets astrocytes (1.5 μl of 10^9^ viral genomes μl^−1^, HANBIO, Shanghai, China) into the hippocampus using the following microinjection coordinates: 2.06 mm caudal of the bregma, ± 1.5 mm lateral from the sagittal midline, and 2 mm deep from the skull surface. Two weeks after microinjection, mice were divided into groups with or without CUS treatment.

### CUS treatment

CUS was used to explore depressive-like behaviors in mice as previously described with some modifications [45, 46]. Mice were exposed to various randomly scheduled, low-intensity social and environmental stressors 2–3 times a day for 4 weeks. The stressors applied as described in Additional file 1: Methods and Materials.

### Behavioral tests

Behavioral tests were conducted after CUS treatment. Behavior was monitored through a video camera positioned in front of the apparatuses, and the images were later analyzed with a Plexon research solutions system (Plexon Inc, Dallas, TX, USA) by an experienced researcher who was blind to the treatment option of the animals tested. Animals completed the SPT, FST, TST, and OFT as described in Additional file 1: Methods and Materials.

### 16S rRNA gene sequencing analysis

The V4-V5 region of the bacteria 16S rRNA gene was detected by PCR. Raw FASTQ files were demultiplexed and quality-filtered using QIIME (version 1.17). Operational taxonomic unit (OTU) were clustered with 97% similarity cutoff using UPARSE (version 7.1 http://drive5.com/uparse/), and chimeric sequences were identified and removed using UCHIME. The phylogenetic affiliation of each 16S rRNA gene sequence was analyzed by RDP Classifier (http://rdp.cme.msu.edu/) against the SILVA (SSU123) 16S rRNA database using a confidence threshold of 70%. To examine dissimilarities in community composition, we performed PCoA in QIIME. More details were described in Additional file 1: Methods and Materials.

### Western blotting (WB) and other experiments

WB was performed as previously described [36]. Real-time PCR, fluorescence in situ hybridization (FISH) in combination with immunostaining, immunostaining, and image analysis were performed as described in the Additional file 1: Methods and Materials.

### Statistical analysis

Statistical analysis was performed using Student’s *t* test, Mann-Whitney test, or one-way analysis of variance (ANOVA) followed by the Holm-Sidak test (SigmaPlot 11.0). The appropriate tests are indicated in figure legends. Results were considered significant at *p* < 0.05. All data were presented as mean ± SEM.

## Additional file

Additional file 1: Supplementary Materials and Methods and Figures S1–S17. (DOCX 3220 kb)

## Data Availability

Raw 16S rRNA reads have been made available on the SRA under accession number SAMN10985890–SAMN10986051.
