# Supplementary material for: Gut microbiota from NLRP3-deficient mice ameliorates depressive-like behaviors by regulating astrocyte dysfunction via circHIPK2
Source: Microbiome. 2019 Aug 22;7:116. doi: 10.1186/s40168-019-0733-3 (PMC6706943; doi:10.1186/s40168-019-0733-3)
Supplement: Supplementary file 1 — Supplementary Materials and Methods and Figures S1–S17. (DOCX 3220 kb) [file 40168_2019_733_MOESM1_ESM.docx]

**Additional file 1**

**Gut microbiota from NLRP3-deficient mice ameliorates depressive-like behaviors by regulating astrocyte dysfunction via circHIPK2**

Yuan Zhang^1†^, Rongrong Huang^1†^, Mengjing Cheng^1†^, Lirui Wang^2^, Jie Chao^3^, Junxu Li^4^, Peng Zheng^5^, Peng Xie^5^, Zhijun Zhang^6^, Honghong Yao^1,7,8*^

^1^Department of Pharmacology, School of Medicine, Southeast University, Nanjing, Jiangsu, China; ^2^School of Basic Medicine and Clinical Pharmacy, China Pharmaceutical University, Nanjing, Jiangsu, China; ^3^Department of Physiology, School of Medicine, Southeast University, Nanjing, Jiangsu, China; ^4^Department of Pharmacology and Toxicology, University at Buffalo, Buffalo, NY, USA; ^5^Department of Neurology, The First Affiliated Hospital of Chongqing Medical University, Chongqing, China; ^6^Department of Neurology of Affiliated ZhongDa Hospital, Institute of Neuropsychiatry of Southeast University, Nanjing, Jiangsu, China; ^7^Institute of Life Sciences, Key Laboratory of Developmental Genes and Human Disease, Southeast University, Nanjing, Jiangsu, China; ^8^Co-innovation Center of Neuroregeneration, Nantong University, Nantong, Jiangsu, China.

† These authors contributed equally to this work.

*To whom correspondence should be addressed: Honghong Yao, Ph.D., Department of Pharmacology, Medical School of Southeast University, Nanjing, 210009, Jiangsu, China; Tel: +8625 83272551; E-mail: yaohh@seu.edu.cn

**Table of Contents**

Supplementary Materials and Methods……………………………………………2

References………………………………………….………………………………11

Supplementary Figures 1-7……………………………………………………….12

**Supplementary Methods and Materials**

**Animals**

The NLRP3 KO mice were kindly shared by Dr. Jurg Tschopp from University of Lausanne and Dr. Rongbin Zhou from Institute of Immunology and the CAS Key Laboratory of Innate Immunity and Chronic Disease, School of Life Sciences and Medical Center, University of Science and Technology of China. NLRP3 KO mice on the C57BL/6 background were described previously [[1](#_ENREF_1)]. The offspring were genotyped by PCR amplification of tail DNA. The following primers were used to amplify a 500 bp product corresponding to the mutant allele and a 250 bp fragment corresponding to the WT allele: mutant 5′-AAGTCGTGCTGCTTCATGT-3′; WT common 5′-TCAAGCTAAGAGAACTTTCTG-3′; and WT 5′-ACACTCGTCATCTTCAGCA-3′. PCR conditions consisted of an initial denaturing at 95 °C for 5 min followed by 40 cycles of 95 °C for 45 s, 55 °C for 30 s, and 72 °C for 40 s.

C57BL/6J mice (male, 6-8 weeks) were purchased from the Model Animal Research Center of Nanjing University (Nanjing, China) and randomly assigned to experimental groups. All animals were housed under conditions of constant temperature and humidity on a 12 h light: 12 h dark cycle, with lights on at 07:00 h. Food and water were available *ad libitum*. Animals were deeply anesthetized via an overdose of isoflurane and then subjected to pneumothorax before they were perfused. All animal procedures were performed in strict accordance with the ARRIVE guidelines, and all animal protocols were approved by the Institutional Animal Care and Use Committee of the Medical School of Southeast University (approval ID: SYXK-2010.4987).

**CUS treatment**

CUS was used to explore depressive-like behaviors in mice as previously described with some modifications [[2](#_ENREF_2), [3](#_ENREF_3)]. Mice were exposed to various randomly scheduled, low-intensity social and environmental stressors 2-3 times a day for 4 weeks. The stressors applied included the following: food deprivation for 24 h, water deprivation for 24 h, overnight illumination, absence of sawdust in cage for 24 h, moistened sawdust with water for 24 h, forced swimming at 8°C for 5 min, tail nipping (1 cm from the tip of the tail), physical restraint for 6 h, and 45° cage-tilt along the vertical axis for 3 h.

**Behavioral tests**

Behavioral tests were conducted after CUS treatment. All tests were carried out between 9:00 and 17:00 h in a sound-attenuated room under low-intensity light and were scored by the same rater. Mice were habituated in the room for at least 3 h before the tests.

**SPT:** Mice were habituated to drinking from one bottle of water and one bottle of 1% sucrose solution for 3 days before testing. After one day of drinking only water, mice were subjected to a 24 h preference test in which water and 1% sucrose solution were delivered from identical bottles. Water and sucrose intake were measured daily, and the positions of the two bottles were switched every 4 h. The bottles were weighed at the start and end of the testing period, and the sucrose preference (%) was calculated as the volume of sucrose intake over the total volume of fluid intake.

**OFT:** Mice were placed in the open field (50 cm × 50 cm) and allowed to explore for 5 minutes. The total distance and the time and distance spent exploring the central region were measured using the ANY-maze Behavioural Tracking System (Stoelting Co., Wood Dale, Illinois).

**FST:** Mice were placed individually in a cylinder (diameter: 20cm; height: 25cm) containing a depth of 15cm 23±1°C water so that mice could not support themselves by touching the bottom with their feet. Water was changed after every trial. A camera positioned directly in front of the cylinder recorded the 6 min swim session. For each test session, the first 2 min served as the habituation period. Immobility time was measured during the last 4 min of the test. Immobility in this test was defined as the absence of any movement except those required keeping the animal’s head above water.

**TST:** The apparatus consisted of a TST box (50 × 50 × 50 cm). Mice were suspended from the ceiling of the box by adhesive tape placed approximately 1 cm below the tip of the tail. In front of the box, a camera was used to record mouse behavior for a 6-min test session. The first 2 min served as the habituation period, and immobility time was measured during the last 4 min. Immobility in this test was defined as the absence of any limb or body movements, except those caused by respiration.

The behaviors of FST and TST were monitored through a video camera positioned in front of the apparatuses, and the images were later analyzed with the ANY-maze Behavioural Tracking System (Stoelting Co., Wood Dale, Illinois) by an experienced researcher who was blind to the treatment option of the animals tested.

**Bacterial DNA extraction and 16S ribosomal RNA (16S rRNA) gene sequencing**

Fecal samples were centrifuged to collect the precipitation, and lysozyme at 20 mg/ml was added. After the suspension was incubated for 1 h at 37°C, proteinase K (20 mg/ml) was added, followed by further incubation at 50°C for 3 h. Subsequently, sodium chloride (5 M) was added, and incubation continued for 10 min at 65°C. DNA purification was performed using both phenol-chloroform-isoamyl alcohol (25:24:1, v/v) extraction and ethanol precipitation. The V4-V5 region of the bacteria 16S rRNA gene was amplified by PCR (95°C for 2 min, followed by 25 cycles at 95°C for 30 s, 55°C for 30 s, and 72°C for 30 s and a final extension at 72°C for 5 min) using primers forward 5’-barcode-GTGCCAGCMGCCGCGG-3’ and reverse 5’-CCGTCAATTCMTTTRAGTTT-3’. An eight-base unique barcode was assigned to each sample. PCR reactions were performed in triplicate 20 μl mixtures containing 4 μl of 5 × FastPfu Buffer, 2 μl of 2.5 mM dNTPs, 0.8 μl of each primer (5 μM), 0.4 μl of FastPfu Polymerase, and 10 ng of template DNA. Amplicons were extracted from 2% agarose gels, purified using the AxyPrep DNA Gel Extraction Kit (Axygen Biosciences, Union City, CA, USA) according to the manufacturer’s instructions and quantified using QuantiFluor™-ST (Promega, USA)

**16S rRNA gene sequencing analysis**

Raw FASTQ files were demultiplexed and quality-filtered using QIIME (version 1.17) with the following criteria: (i) 250 bp reads were truncated at any site receiving an average quality score <20 over a 10 bp sliding window, and truncated reads that were shorter than 50 bp were discarded. (ii) Barcodes must match exactly. Two nucleotides in primer mismatching and reads containing ambiguous characters were removed. (iii) Only sequences that overlap longer than 10 bp were assembled according to their overlap sequences. Reads that could not be assembled were discarded.

Operational taxonomic unit (OTU) were clustered with 97% similarity cutoff using UPARSE (version 7.1 http://drive5.com/uparse/), and chimeric sequences were identified and removed using UCHIME. The phylogenetic affiliation of each 16S rRNA gene sequence was analyzed by RDP Classifier (http://rdp.cme.msu.edu/) against the SILVA (SSU123) 16S rRNA database using a confidence threshold of 70%. To examine dissimilarities in community composition, we performed Principal Coordinates Analysis (PCoA) in QIIME. In PCoA, a distance matrix is used to plot n samples in (n−1)-dimensional space. The result was used to compare groups of samples based on unweighted UniFrac distance metrics.

**Western blot analysis**

Proteins were extracted in RIPA lysis buffer (P0013B, Beyotime, Shanghai, China). Proteins were then separated by sodium dodecyl sulfate-polyacrylamide gel electrophoresis and transferred to polyvinylidene fluoride membranes. The membranes were blocked with 5% non-fat dry milk in Tris-buffered saline with Tween-20 and then incubated overnight at 4°C with the following primary antibodies: anti-GFAP (1:2000, G3893) obtained from Sigma-Aldrich (St. Louis, MO, USA) and anti-GAPDH (1:2000, 60004) acquired from Proteintech (Wuhan, China). The horseradish peroxidase-conjugated goat anti-mouse (ZB5305, ZSGB-BIO, Beijing, China) or rabbit (ZB5301, ZSGB-BIO, Beijing, China) IgG secondary antibody (1:2000) was incubated for 1 h. The proteins were then detected by a MicroChemi 4.2^®^ (DNR, Israel) digital image scanner. Band intensity was quantified using Image J software (NIH, USA).

**Real-time PCR**

Total RNA was extracted from the brain tissues of mice using Trizol reagent (9109, TAKARA, Kusatsu, Shiga, Japan) and the plasma of mice using miRNeasy Serum/Plasma Kit (217184, QIAGEN, Duesseldorf, Germany). CircRNAs were reverse transcribed using the HiScript Q RT SuperMix for qPCR Kit (R123-01, Vazyme, Nanjing, China) and quantified via SYBR Green real-time PCR (Q141-02, Vazyme, Nanjing, China). The results were standardized to the control values of 18S. The following primers were employed: mouse divergent circHIPK2 (forward: 5’-GACAACCGTACCGAGTGAAG-3’; reverse: 5’-GTGTGAGGGGAGAAAACTTGC-3’), mouse circHIPK2 convergent primer (forward: 5’-GCAAGTTTTCTCCCCTCACAC-3’; reverse: 5’-CTTCACTCGGTACGGTTGTC-3’), mouse divergent GAPDH primer (forward: 5’-AGGTCGGTGTGAACGGATTTG-3’; reverse: 5’-GGGGTCGTTGATGGCAACA-3’), and mouse GAPDH convergent primer (forward: 5’-AGGTCGGTGTGAACGGATTTG-3’; reverse: 5’-GGGGTCGTTGATGGCAACA-3’).

**Fluorescence in situ hybridization (****FISH)** **in combination with immunostaining**

Based on our previous study [[4](#_ENREF_4)], 30 μm sections encompassing the entire hippocampus were cut on a cryostat. The sections were fixed with 4% paraformaldehyde for 20 min, permeabilized with 0.25% Triton X-100 (T109027, Aladdin, Shanghai, China) in PBS for 15 min, and prehybridized in a hybridization buffer (50% formamide, 10 mM Tris-HCl, pH 8.0, 200 mg/ml yeast tRNA [15401–011, Sigma-Aldrich, St. Louis, MO, USA], 1X Denhardt solution [30915, Sigma-Aldrich, St. Louis, MO, USA], 600 mM NaCl, 0.25% SDS [15553–035, Invitrogen, Shanghai, China], 1 mM EDTA, and 10% dextran sulfate [D8906, Sigma-Aldrich, St. Louis, MO, USA]) for 1 h at 37°C. A hybridization buffer containing 500 nM of a commercially available biotin-labeled circHIPK2 probe (Invitrogen, Shanghai, China) was preheated to 65°C for 5 min. Hybridization occurred at 37°C overnight. The next day, sections were washed three times in 2XSSC (10% v/v 20XSSC [15557, Invitrogen, Shanghai, China] in DEPC-treated water [V900882, Vetec, Beijing, China]) and twice in 0.2XSSC (10% v/v 2XSSC in DEPC-treated water) at 42°C. After blocking with 1% BSA (BS043D, Biosharp, Hefei, China) and 3% normal goat serum (ZLI-0956, ZSGB-BIO, Beijing, China) in PBS for 1 h at room temperature, sections were incubated with a FITC-streptavidin (1:200, 434311, Life Technology, Shanghai, China) overnight at 4°C. The third day, sections were washed three times with TBS (0.1 M Tris, 0.308 M NaCl, pH 7.4), blocked for 1h at room temperature in 1% BSA and 1% Triton X-100 in PBS (wt/vol for BSA and vol/vol for Triton X-100), and then incubated with the primary antibody GFAP (1:600, G3893, Sigma-Aldrich, St. Louis, MO, USA) overnight at 4°C. The final day, sections were incubated with Alexa Fluor 594 goat anti-mouse IgG (1:250, A11005, Invitrogen, Shanghai, China) in PBS for 1 h at room temperature and mounted with Prolong Gold Anti-fade reagent containing DAPI (0100-20, SouthernBiotech, Birmingham, AL, USA) for visualization of nuclei. Immunofluorescence images were captured by microscopy (Olympus DP73, Olympus, Tokyo, Japan).

**Immunostaining and image analysis**

Based on our previous study [[5](#_ENREF_5)], 30 μm sections encompassing the entire hippocampus were cut on a cryostat. The sections were permeabilized with 0.3% Triton X-100 in PBS for 15 min and blocked with 10% NGS in 0.3% Triton X-100 for 1 h at room temperature. The sections were then incubated with a mouse anti-GFAP antibody (1:600, G3893, Sigma-Aldrich, St. Louis, MO, USA) overnight at 4°C. The next day, the sections were washed and incubated with Alexa Fluor 594 goat anti-mouse IgG (1:250, A11005, Invitrogen, Shanghai, China) in PBS for 1 h at room temperature. After a final washing with PBS, the sections were mounted onto glass slides, and ProLong Gold Anti-fade reagent containing DAPI (0100-20, SouthernBiotech, Birmingham, AL, USA) was applied for visualization of nuclei. Immunofluorescence images were captured by microscopy (Olympus DP73, Olympus, Tokyo, Japan). Average intensities of GFAP were calculated using Image J software (NIH, USA) by sampling a 28×28 pixel area and capturing 36 images from 6 consecutive sections. The values were reported as the average intensity above the background±SD. Computer-based cell tracing software Neurolucida 360 (MBF Bioscience, Williston, VT, USA) was used for three-dimensional (3D) reconstruction of GFAP positive cells within the hippocampus. NeuroExplorer (MBF Bioscience, Williston, VT, USA) was used to analyze 10 cells per animal. Sholl analysis was used to determine branch tree morphology by placing three-dimensional concentric circles in 5 mm increments starting at 5 mm from the soma.

**References**

1. Martinon F, Petrilli V, Mayor A, Tardivel A, Tschopp J: Gout-associated uric acid crystals activate the NALP3 inflammasome. Nature. 2006; 440**:**237-241.

2. Barthas F, Humo M, Gilsbach R, Waltisperger E, Karatas M, Leman S, Hein L, Belzung C, Boutillier AL, Barrot M, Yalcin I: Cingulate Overexpression of Mitogen-Activated Protein Kinase Phosphatase-1 as a Key Factor for Depression. Biol Psychiatry. 2017; 82**:**370-379.

3. Willner P, Muscat R, Papp M: Chronic mild stress-induced anhedonia: a realistic animal model of depression. Neurosci Biobehav Rev. 1992; 16**:**525-534.

4. Zhang Y, Shen K, Bai Y, Lv X, Huang R, Zhang W, et al: Mir143-BBC3 cascade reduces microglial survival via interplay between apoptosis and autophagy: Implications for methamphetamine-mediated neurotoxicity. Autophagy. 2016; 12**:**1538-1559.

5. Yao H, Ma R, Yang L, Hu G, Chen X, Duan M, et al: MiR-9 promotes microglial activation by targeting MCPIP1. Nature communications. 2014; 5**:**4386.

**Supplementary Figures**

**
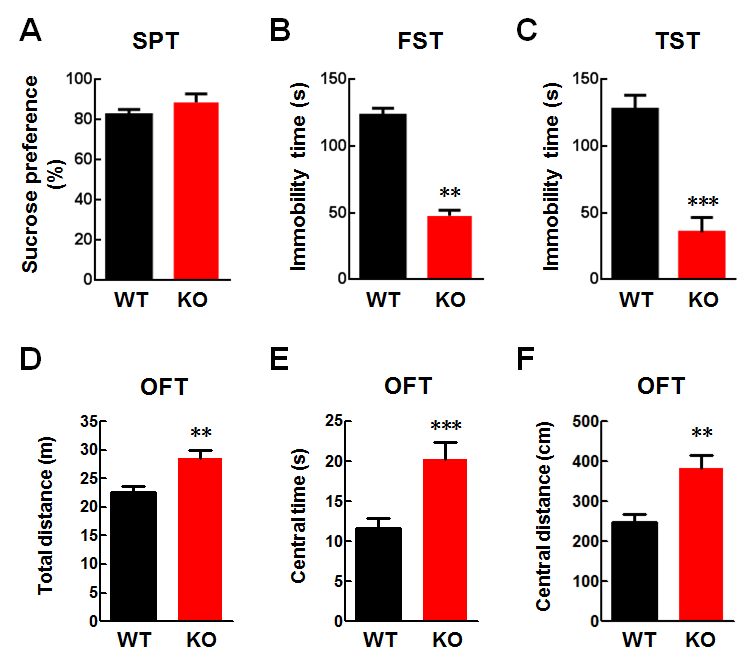
**

**Figure S1.** **NLRP3 inflammasome deficiency affected mood-related behavior.** **(A)** NLRP3 KO mice displayed no difference in sucrose preference compared with WT mice. **(B-D)** NLRP3 KO mice displayed decreased immobility time in the FST **(B)** and TST **(C)**, and increased total distance **(D)**, time **(E)** and distance **(F)** traveled in the central area of the OFT compared with WT mice. N=9-15 mice/group. **p<0.01 and ***p<0.001 vs. the WT group using Student’s t-test.

**
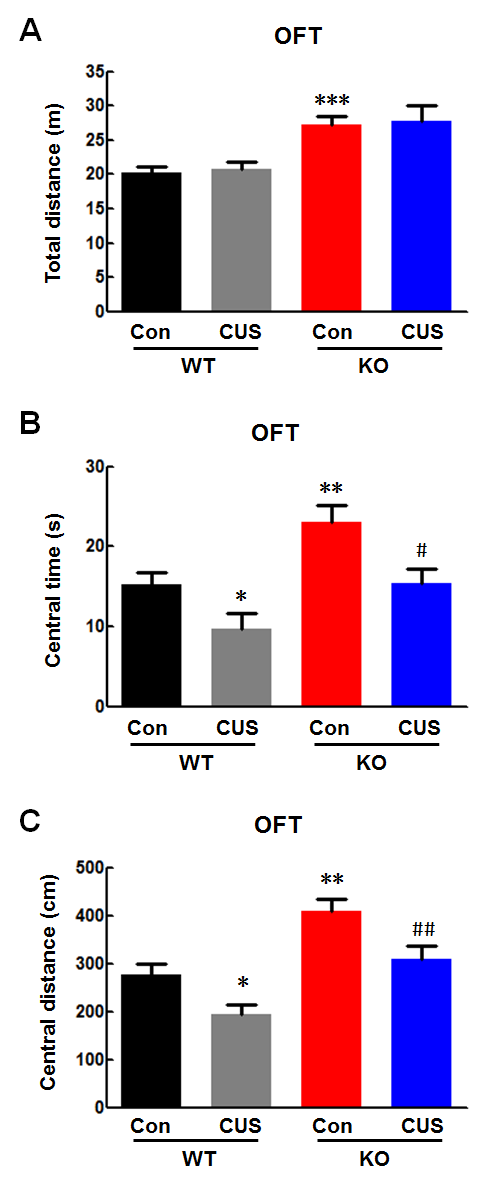
**

**Figure S2.** **NLRP3 inflammasome deficiency** **affected mood-related behavior. (A)** There was no influence of CUS treatment on locomotor activity, but NLRP3 inflammasome deficiency increased the total distance in the OFT. **(B-C)** Compared with WT mice, NLRP3 inflammasome deficiency inhibited the decreased time **(B)** and distance **(C)** spent exploring the central region in the OFT induced by CUS. N=6-13 mice/group. *p<0.05, **p<0.01 and ***p<0.001 vs. the WT control group. ^#^p<0.05 and ^##^p<0.01 vs. the CUS-treated WT group using one-way ANOVA followed by the Holm-Sidak test.

**
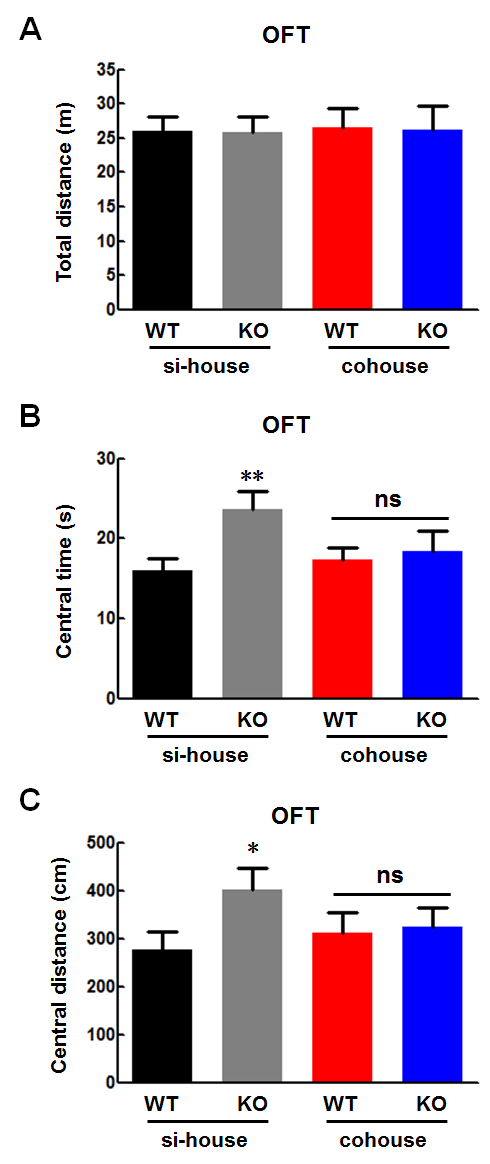
**

**Figure S3.** **Cohousing affected mood-related behavior.** **(A)** Cohousing exerted no significant effect on the total distance in the OFT. **(B-C)** Cohousing reduced the significant differences in the time **(B)** and distance **(C)** spent exploring the central region in the OFT between WT and NLRP3 KO littermates. N=11 mice/group. *p<0.05 and **p<0.01 vs. the si-house-treated WT group using Student’s t-test. si-house: only WT mice or KO mice were housed in a cage. cohouse: WT and KO mice were housed in a cage.

**
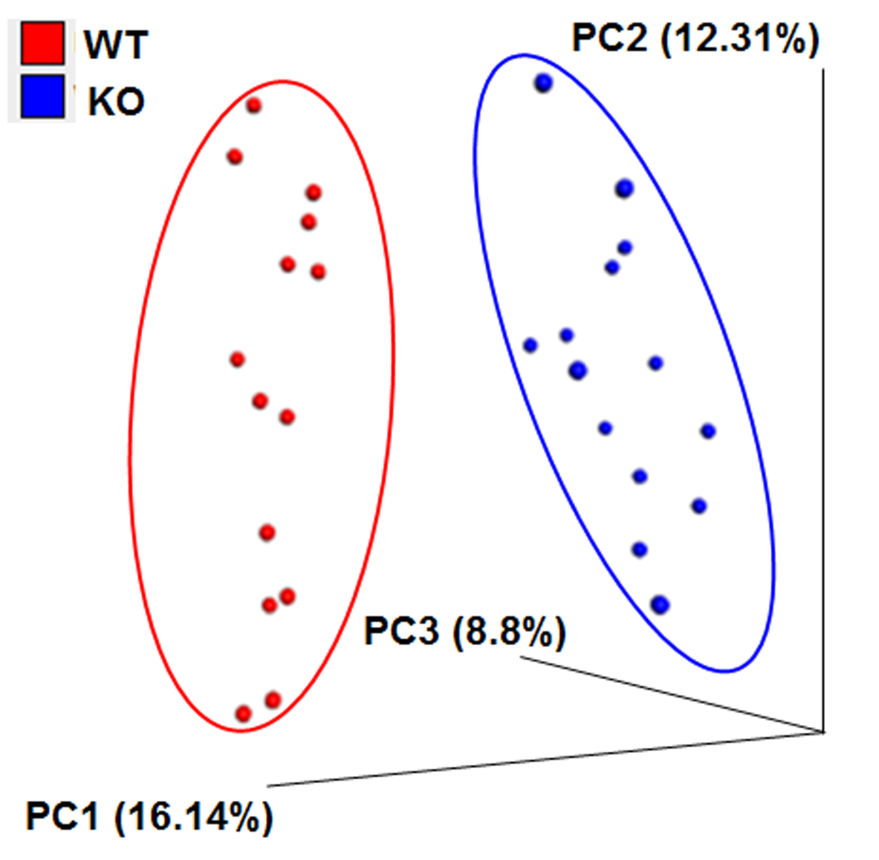
**

**Figure S4. Distinct microbial landscape between the WT and NLRP3 KO groups.** Three-dimensional PCoA of unweighted UniFrac distances showed obvious differences in the gut microbiota composition between WT and NLRP3 KO mice.

**
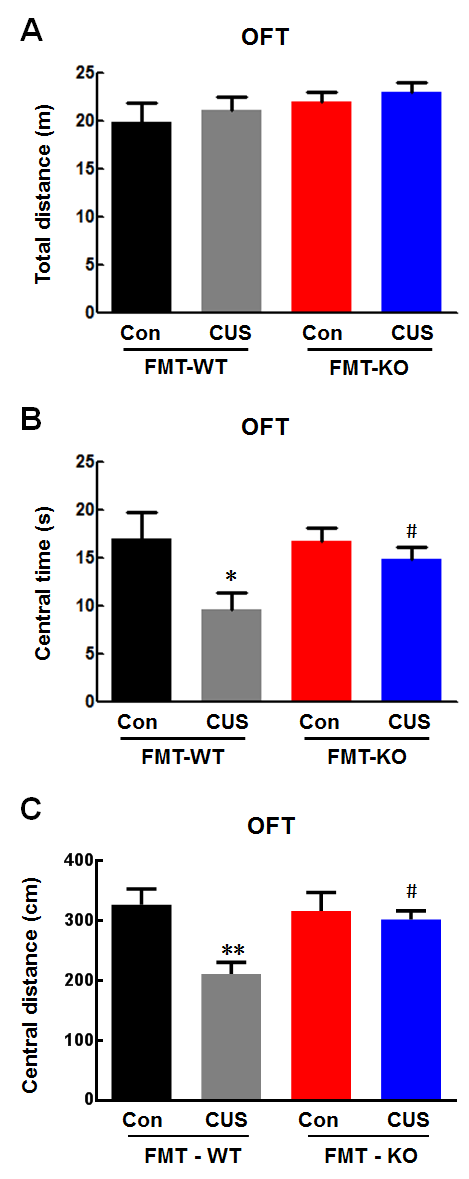
**

**Figure S5.** **FMT affected mood-related behavior. (A)** FMT exerted no significant effect on the total distance in the OFT. **(B-C)** Compared to WT microbiota recipient mice, NLRP3 KO microbiota recipient mice displayed an inhibition of the CUS-induced decreases in the time **(B)** and distance **(C)** spent exploring the central region in the OFT. N=14 mice/group. *p<0.05 and **p<0.01 vs. the FMT-WT control group. ^#^p<0.05 vs. the CUS-treated FMT-WT group using one-way ANOVA followed by the Holm-Sidak test.

**
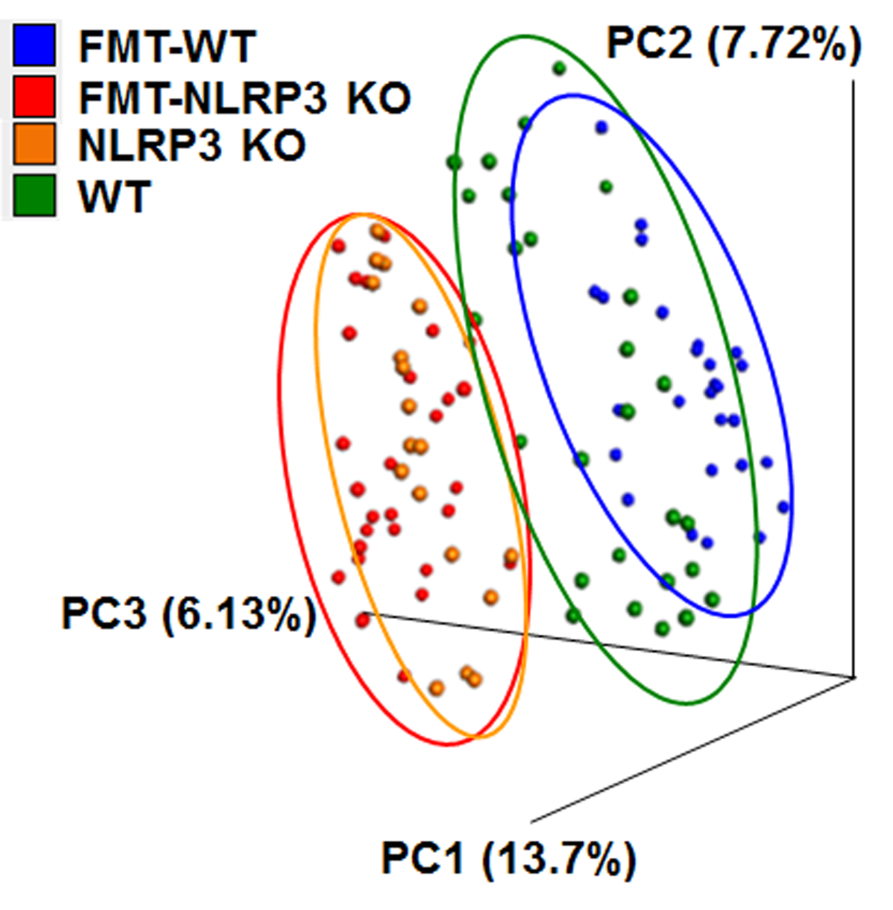
**

**Figure S6. The gut microbiome was successfully transplanted in the FMT experiment.** Three-dimensional PCoA of unweighted UniFrac distances showed a clear difference between the WT microbiota recipient mice and the NLRP3 KO microbiota recipient mice, while there similarities were observed between corresponding donor and recipient mice.


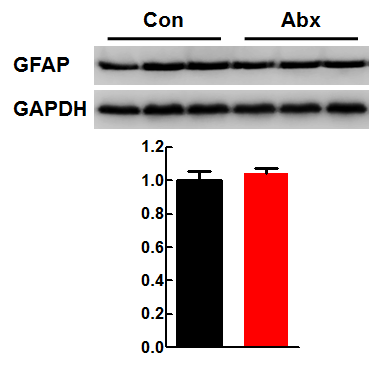


**Figure S7.** **Impact of the antibiotic cocktail on astrocyte activation.** Antibiotic cocktail treatment did not influence on GFAP expression. n=3-5 mice/group.


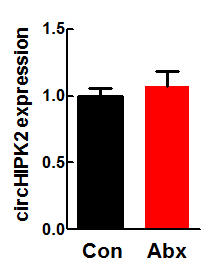


**Figure S8.** **Impact of the antibiotic cocktail on circHIPK2 expression.** Antibiotic cocktail treatment did not exert significant effect on the expression of circHIPK2. n=3-5 mice/group.


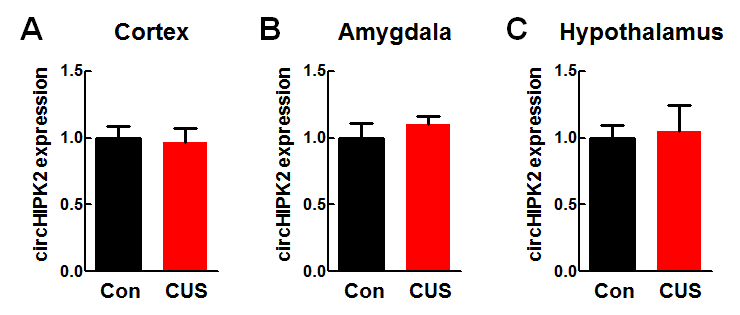


**Figure S9.** **circHIPK2 expression in different brain regions.** **(A-C)** The circHIPK2 expression in brain regions, such as the cortex **(A)**, amygdala **(B)** and hypothalamus **(C)**, isolated from the CUS-treated group was not significantly different from that in the control group. n=6 mice/group.


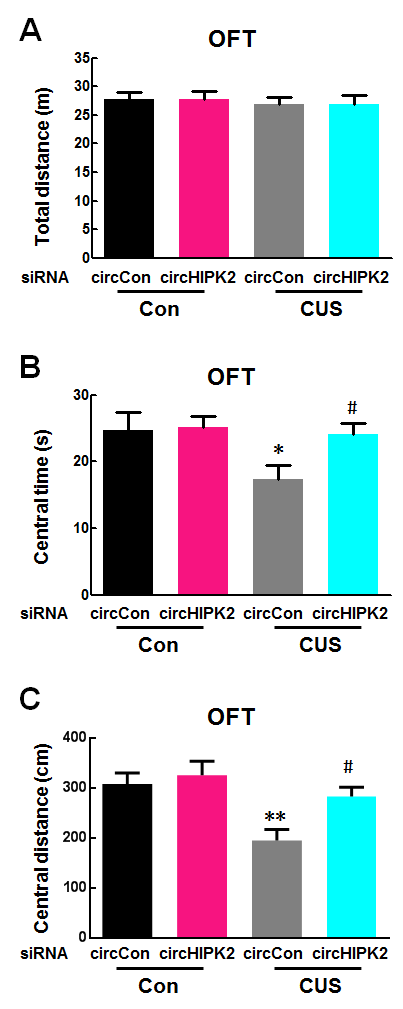


**Figure S10.** **circHIPK2 siRNA microinjection affected mood-related behavior. (A)** circHIPK2 siRNA microinjection exerted no significant effect on the total distance in the OFT. **(B-C)** circHIPK2 siRNA microinjection significantly inhibited the CUS-induced decreases in the time **(B)** and distance **(C)** spent exploring the central region in the OFT. N=13 mice/group. *p<0.05, and **p<0.01 vs. the circCon control group. ^#^p<0.05 vs. the CUS-treated circCon group using one-way ANOVA followed by the Holm-Sidak test.


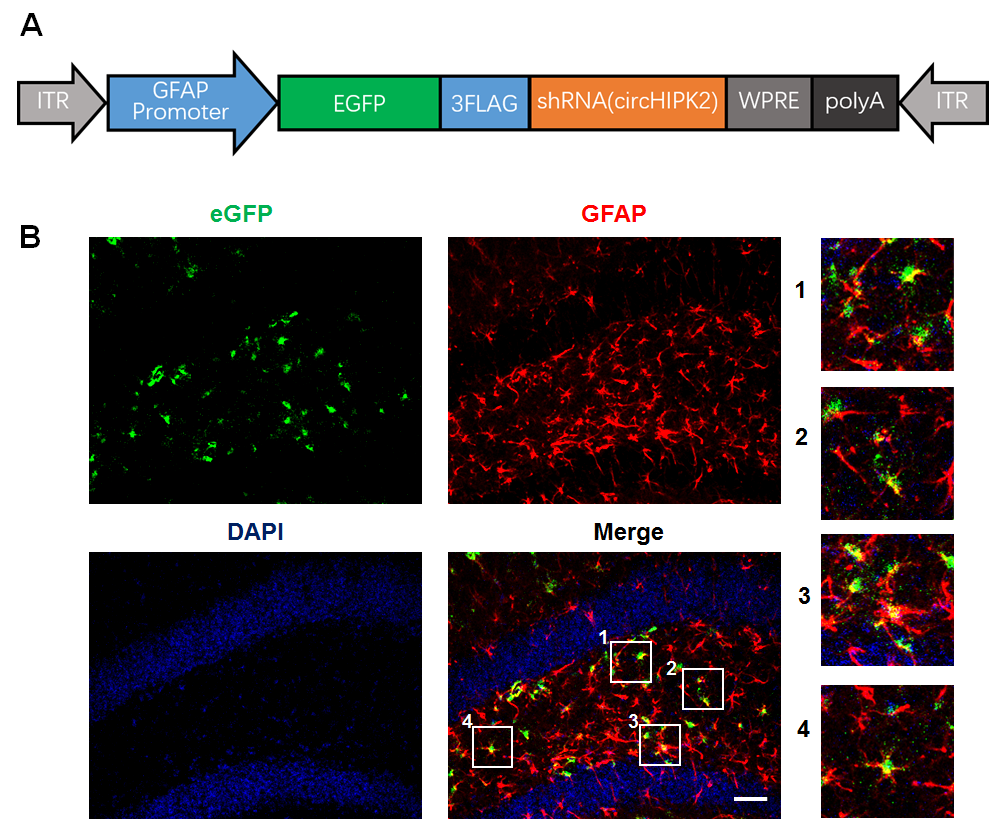


**Figure S11. Efficiency of astrocyte-specific AAVs in hippocampi.** **(A)** Schematic representation showing mouse circHIPK2 subcloned into an AAV plasmid under transcriptional regulation of the GFAP promoter (AAV-EGFP-3FLAG-circHIPK2). An AAV-EGFP-3FLAG plasmid that did not encode circHIPK2 served as the control. **(B)** Representative images of mouse hippocampi microinjected with AAVs. Green: eGFP; Red: GFAP; Blue: DAPI. Scale bar: 50 μm.


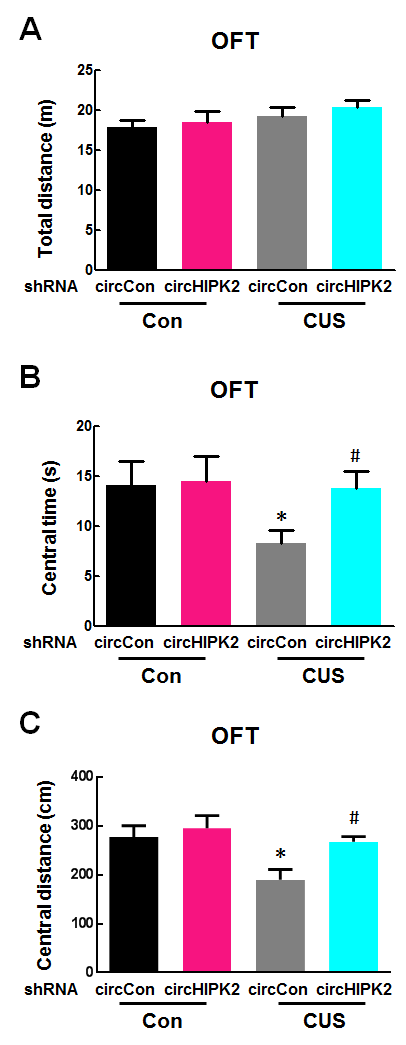


**Figure S12.** **Specific knockdown of circHIPK2 expression in astrocytes affected mood-related behavior. (A)** Specific knockdown of circHIPK2 expression in astrocytes exerted no significant effect on the total distance in the OFT. **(B-C)** Specific knockdown of circHIPK2 expression in astrocytes significantly inhibited the CUS-induced decreases in the time **(B)** and distance **(C)** spent exploring the central region in the OFT. N=10 mice/group. *p<0.05 vs. the circCon control group. ^#^p<0.05 vs. the CUS-treated circCon group using one-way ANOVA followed by the Holm-Sidak test.


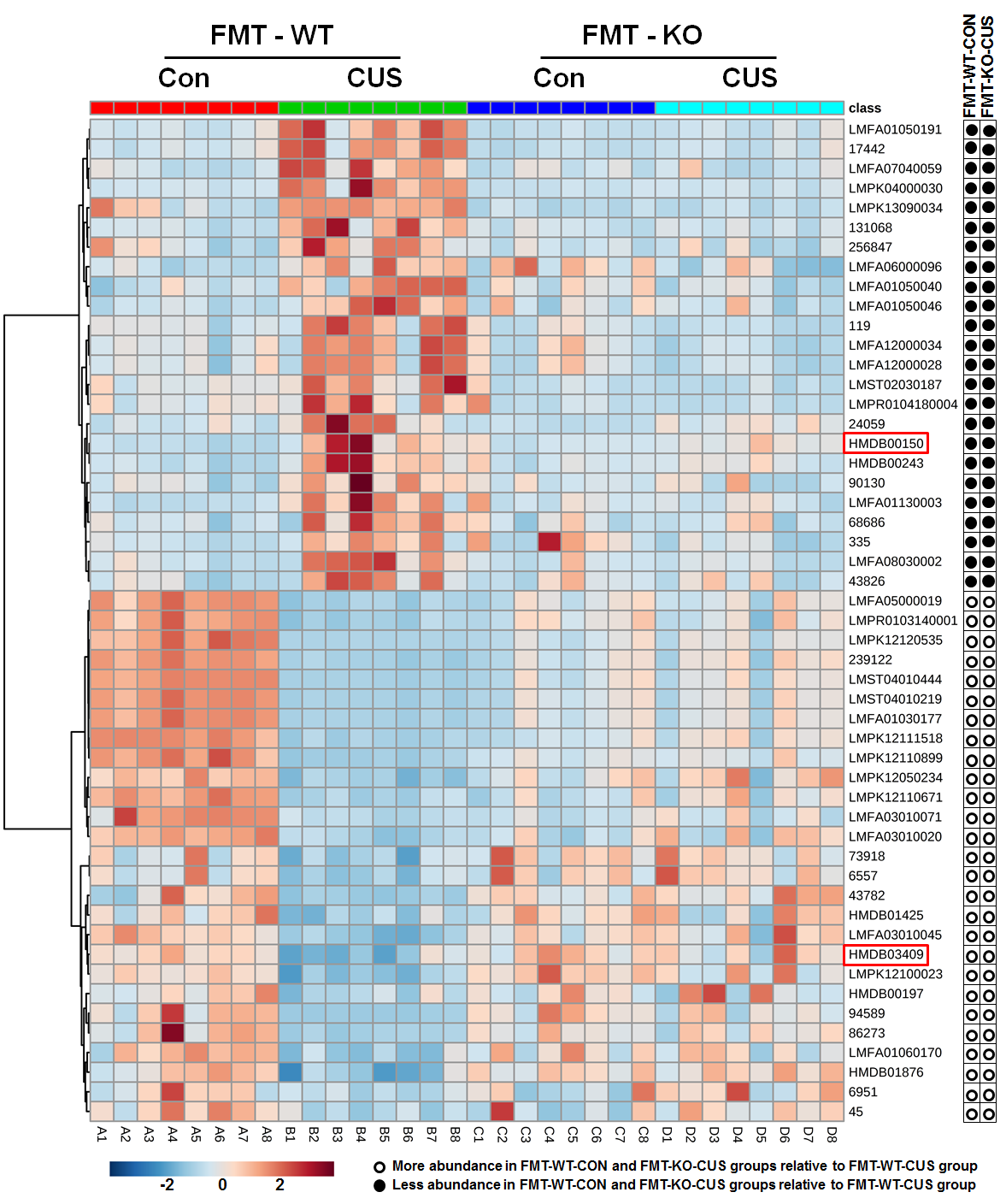


**Figure S13. Heatmap of identified differential metabolites with fecal metabolomics profile.** Each cell in the heatmap represents the fold change of a particular metabolite.

**
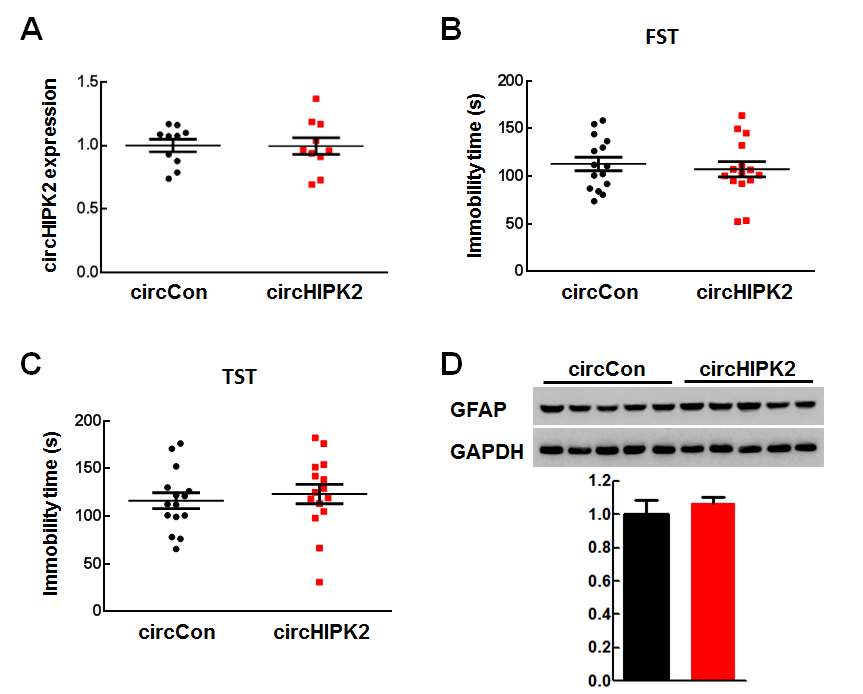
**

**Figure S14. Impact of the intravenous injection of** **circHIPK2 on depressive-like behavior or astrocyte activation.** **(A)** The intravenous injection of circHIPK2 could not affect the expression of circHIPK2 in the brain. n=10 mice/group. **(B-C)** The intravenous injection of circHIPK2 did not affect the immobility time in the FST **(B)** and TST **(C)**, n=15 mice/group. **(D)** The intravenous injection of circHIPK2 did not affect GFAP expression compared with that in the circ-control group. n=10 mice/group.


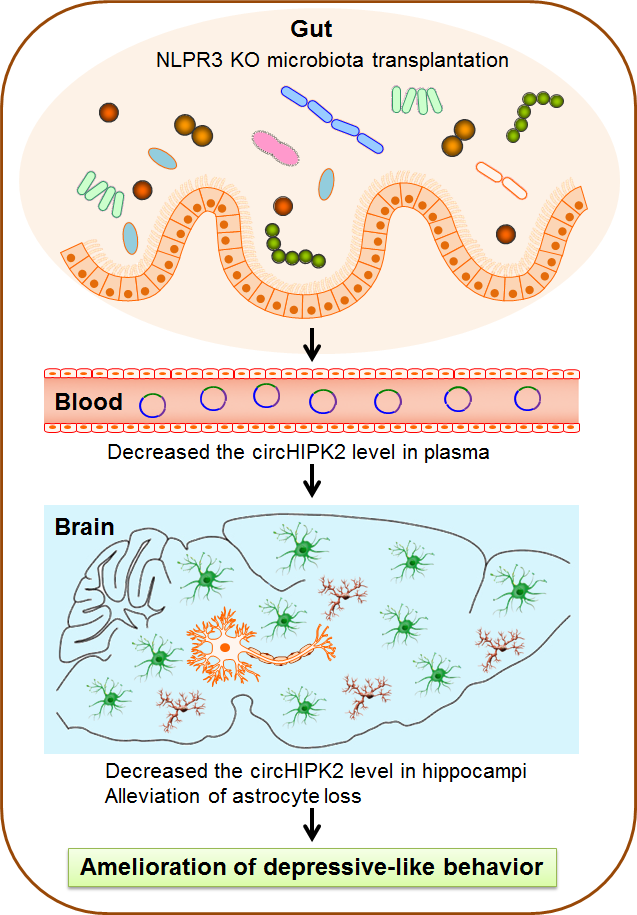


**Figure S15.** **Gut microbiota from NLRP3 KO mice ameliorated depressive-like behaviors by regulating astrocyte dysfunction via circHIPK2.** Transplantation of the gut microbiota from NLRP3 KO mice significantly decreased the levels of circHIPK2 in plasma and hippocampi with consequent alleviation of astrocyte loss, resulting in amelioration of the depressive-like behavior induced by CUS.


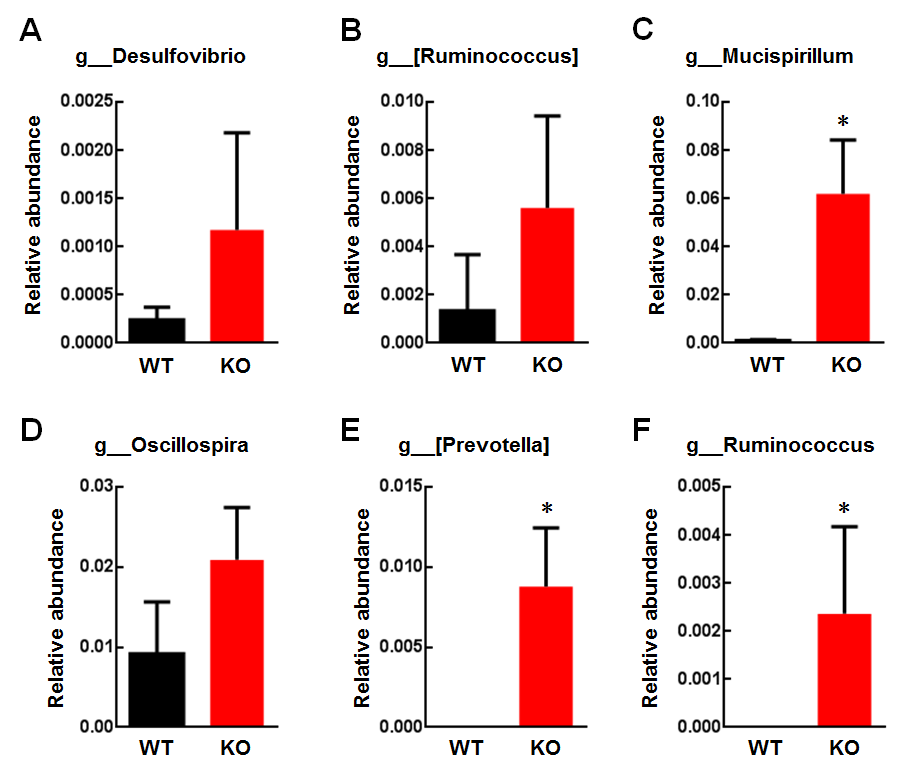


**Figure S16. Caspase-1 deficiency affected the gut microbiota composition.** **(A-F)** Relative abundance changes of genera in the gut microbiota of caspase-1 KO littermates. N=4 mice/group. *p<0.05 vs. the WT group using the Mann-Whitney test.


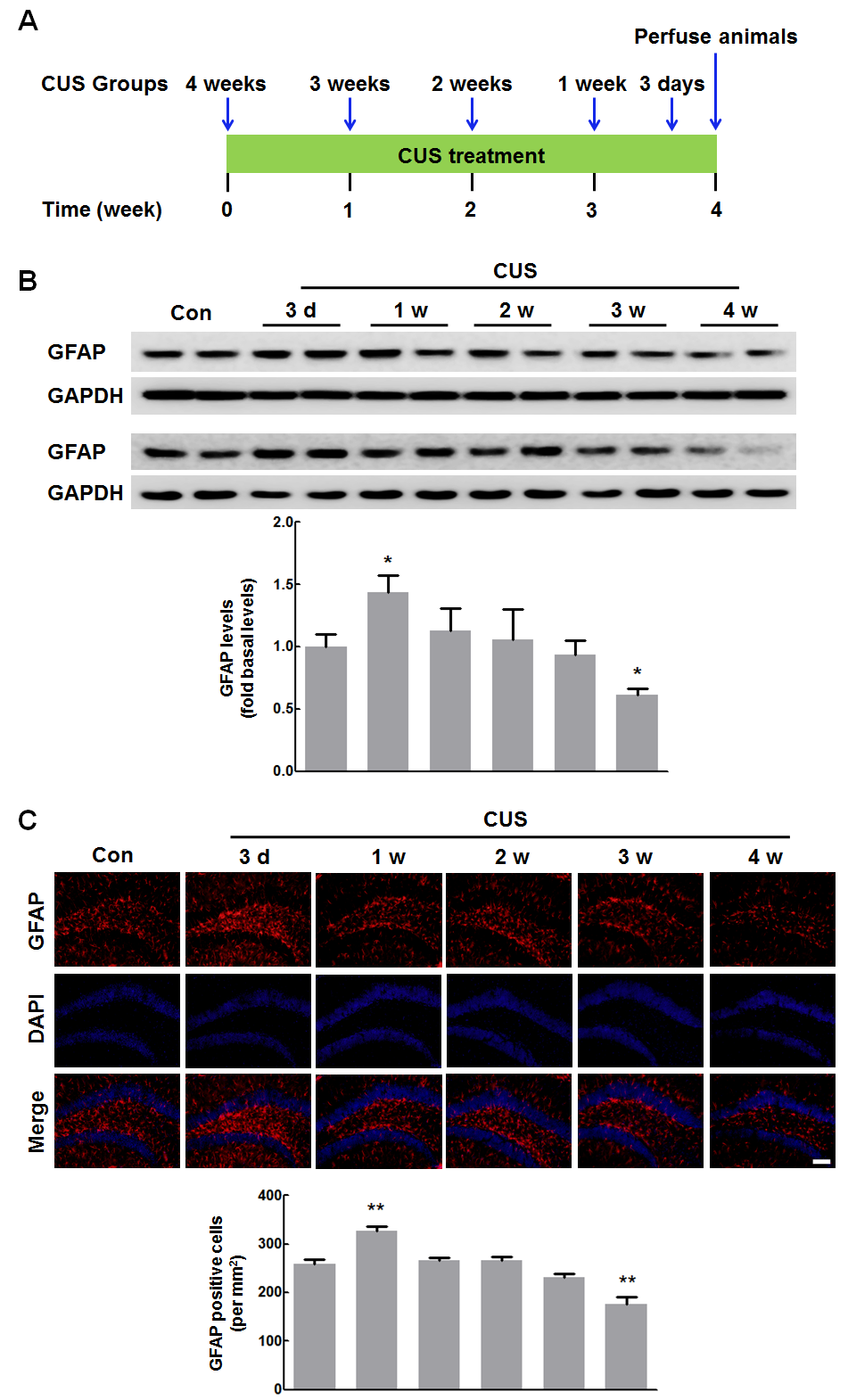


**Figure S17.** **Dynamic changes in GFAP expression during the process of CUS.** **(A)** Illustration of the CUS experimental procedure. Mice were treated with CUS for 4 weeks, 3 weeks, 2 weeks, 1 week, and 3 days before being sacrificed. **(B)** GFAP expression in mouse hippocampi. Mice were treated with CUS for different time points (3 days, 1 week, 2 weeks, 3 weeks, and 4 weeks), followed by the detection of GFAP expression by western blotting. N=4 mice/group. **(C)** Representative images of GFAP immunostaining for different time points (3 days, 1 week, 2 weeks, 3 weeks, and 4 weeks) in mouse hippocampi. Scale bars: 100 μm. Quantification of GFAP positive cells per mm^2^ in mouse hippocampi. N=3 mice/group. *p<0.05 and **p<0.01 vs. the WT group using Student’s t-test.
